# TIE1 and TEK signalling, intraocular pressure, and primary open-angle glaucoma: a Mendelian randomization study

**DOI:** 10.1186/s12967-023-04737-9

**Published:** 2023-11-24

**Authors:** Skanda Rajasundaram, Nazlee Zebardast, Puja Mehta, Anthony P. Khawaja, Alasdair Warwick, Katherine Duchinski, Stephen Burgess, Dipender Gill, Ayellet V. Segrè, Janey Wiggs

**Affiliations:** 1 Faculty of Medicine, Imperial College London, London, UK; 2 Department of Ophthalmology, Harvard Medical School, Boston, MA USA; 3 Ocular Genomics Institute, Massachusetts Eye and Ear, Boston, MA USA; 4 Broad Institute of MIT and Harvard, Cambridge, MA USA; 5 NIHR Biomedical Research Centre, Moorfields Eye Hospital NHS Foundation Trust and UCL Institute of Ophthalmology, London, UK; 6 UCL Institute of Cardiovascular Science, London, UK; 7 MRC Biostatistics Unit, University of Cambridge, Cambridge, UK; 8 Department of Epidemiology and Biostatistics, School of Public Health, Imperial College London, London, UK

**Keywords:** Intraocular pressure, Primary open-angle glaucoma, Mendelian randomization, Colocalization, TIE1, TEK

## Abstract

**Background:**

In primary open-angle glaucoma (POAG), lowering intraocular pressure (IOP) is the only proven way of slowing vision loss. Schlemm’s canal (SC) is a hybrid vascular and lymphatic vessel that mediates aqueous humour drainage from the anterior ocular chamber. Animal studies support the importance of SC endothelial angiopoietin-TEK signalling, and more recently TIE1 signalling, in maintaining normal IOP. However, human genetic support for a causal role of TIE1 and TEK signalling in lowering IOP is currently lacking.

**Methods:**

GWAS summary statistics were obtained for plasma soluble TIE1 (sTIE1) protein levels (*N* = 35,559), soluble TEK (sTEK) protein levels (*N* = 35,559), IOP (*N* = 139,555) and POAG (*N*_*cases*_ = 16,677, *N*_*controls*_ = 199,580). Mendelian randomization (MR) was performed to estimate the association of genetically proxied TIE1 and TEK protein levels with IOP and POAG liability. Where significant MR estimates were obtained, genetic colocalization was performed to assess the probability of a shared causal variant (PP_shared_) versus distinct (PP_distinct_) causal variants underlying TIE1/TEK signalling and the outcome. Publicly available single-nucleus RNA-sequencing data were leveraged to investigate differential expression of *TIE1* and *TEK* in the human ocular anterior segment.

**Results:**

Increased genetically proxied TIE1 signalling and TEK signalling associated with a reduction in IOP (− 0.21 mmHg per SD increase in sTIE1, 95% CI = − 0.09 to − 0.33 mmHg, *P* = 6.57 × 10^–4^, and − 0.14 mmHg per SD decrease in sTEK, 95% CI = − 0.03 to − 0.25 mmHg, *P* = 0.011), but not with POAG liability. Colocalization analysis found that the probability of a shared causal variant was greater for TIE1 and IOP than for TEK and IOP (PP_shared_/(PP_distinct_ + PP_shared_) = 0.98 for TIE1 and 0.30 for TEK). In the anterior segment, *TIE1* and *TEK* were preferentially expressed in SC, lymphatic, and vascular endothelium.

**Conclusions:**

This study provides novel human genetic support for a causal role of both TIE1 and TEK signalling in regulating IOP. Here, combined evidence from *cis-*MR and colocalization analyses provide stronger support for TIE1 than TEK as a potential IOP-lowering therapeutic target.

**Supplementary Information:**

The online version contains supplementary material available at 10.1186/s12967-023-04737-9.

## Introduction

Primary open-angle glaucoma is a chronic, degenerative optic neuropathy and represents the leading cause of irreversible blindness worldwide [1, 2]. No curative therapies exist for POAG and so there is a clinical imperative to identify novel efficacious drug targets for its treatment. Pathophysiologically, POAG is characterised by a rise in IOP following increased resistance to aqueous humour drainage from the anterior chamber of the eye, primarily via the trabecular meshwork and Schlemm’s canal (SC), a large lymphatic-like vessel [1]. IOP is an established causal risk factor for POAG and lowering IOP remains the only proven way of slowing visual decline in POAG. Although the involvement of SC in regulating IOP is established, pharmacological therapies targeting SC have yet to be approved for use in clinical practice.

TIE1/TEK signalling in SC has been proposed as a potential therapeutic target in the treatment of elevated IOP [3, 4]. TIE1 and TEK (also known as TIE2) are transmembrane protein receptor tyrosine kinases that are highly expressed in SC endothelial cells [5]. Angiopoietin 1 (ANGPT1) and Angiopoietin 2 (ANGPT2) act as strong and weak agonists, respectively, for the TEK receptor in SC [6]. No direct ligands have been discovered for TIE1 and so it is considered an orphan receptor that interacts functionally with TEK receptor signalling [7, 8]. Historically, animal studies have highlighted the importance of TEK signalling [9–11], but recent evidence suggests that TIE1 signalling may also be critical in the development of SC and the maintenance of normal IOP [3]. In humans, previous GWAS studies have identified significant associations between variants in the *ANGPT1* and *ANGPT2* gene regions with both IOP [12] and POAG [13] but no GWAS associations have been identified in the *TIE1* or *TEK* gene regions. One study highlighted the importance of rare loss-of-function *TEK* mutations in the development of primary congenital glaucoma but no human genetic evidence for TIE1 signalling in the regulation of IOP has yet been shown. Given the high false discovery rate of animal studies in predicting drug target efficacy in humans, human-centric genetic support for TIE1/TEK signalling should be considered critical in its prioritization as a drug target worth pursuing in large-scale randomized controlled trials (RCTs) [14].

Drug target Mendelian randomization leverages naturally arising human genetic variation to infer the causal effect of a putative drug target on an outcome [15, 16]. Here, *‘cis’* genetic variants located in the vicinity of a gene encoding a protein drug target of interest are used to proxy the pharmacological perturbation of this same drug target. The random allocation of genetic variants from parents to offspring is analogous to random treatment allocation in an RCT and thus, the phenotypic effect of a particular genetic variant should not systematically relate to environmental confounding. Germline genetic variation is non-modifiable and temporally precedes the onset of disease, thus the phenotypic effect of a particular genetic variant is also less susceptible to reverse causation. Given the inherent vulnerability of conventional observational studies to unmeasured confounding and reverse causation, MR can strengthen causal inferences made from observational data. Empirical studies show that supportive genetic evidence increases the probability of successful approval of novel drug targets by twofold [17] and that the impact of prior genetic support is strongest when genetic variants from protein-coding regions of the genome are used [18]. Accordingly, leveraging *cis* variants that associate with protein expression levels, so-called protein quantitative trait loci (pQTLs), strengthens the use of MR as a tool to investigate putative drug target efficacy.

Genetic variants associated with increased or decreased protein expression of a given receptor can be considered analogous to lifelong exposure to a drug stimulating or inhibiting this same receptor [19]. We sought to mimic the effect of pharmacologically perturbing membrane-bound TIE1 and TEK receptor signalling (i.e., the effect of this signalling pathway in SC) using summary genetic association data for circulating protein levels of sTIE1 and sTEK. sTIE1 is generated upon proteolytic cleavage of the extracellular domain of membrane-bound TIE1 receptor [20] and such TIE1 ectodomain shedding amplifies membrane-bound angiopoietin-TEK signalling [7, 8]. We therefore assumed a positive relationship between circulating sTIE1 levels and membrane-bound TIE1/TEK signalling. Similarly, sTEK is generated from proteolytic cleavage of the extracellular domain of membrane-bound TEK receptors [21, 22]. However, sTEK contains the ligand-binding domain of the receptor and so it binds angiopoietins, inhibiting the activation and phosphorylation of membrane-bound TEK receptors [22, 23]. We therefore assumed a negative relationship between circulating sTEK levels and membrane-bound TEK receptor signalling. Thus, leveraging large-scale summary-based genetic association data for plasma sTIE1 and sTEK protein levels, we used *cis-*MR and colocalization to infer the causal effect of TIE1 and TEK signalling perturbation on IOP and liability to POAG.

## Methods

### Study design

**Fig. 1:**
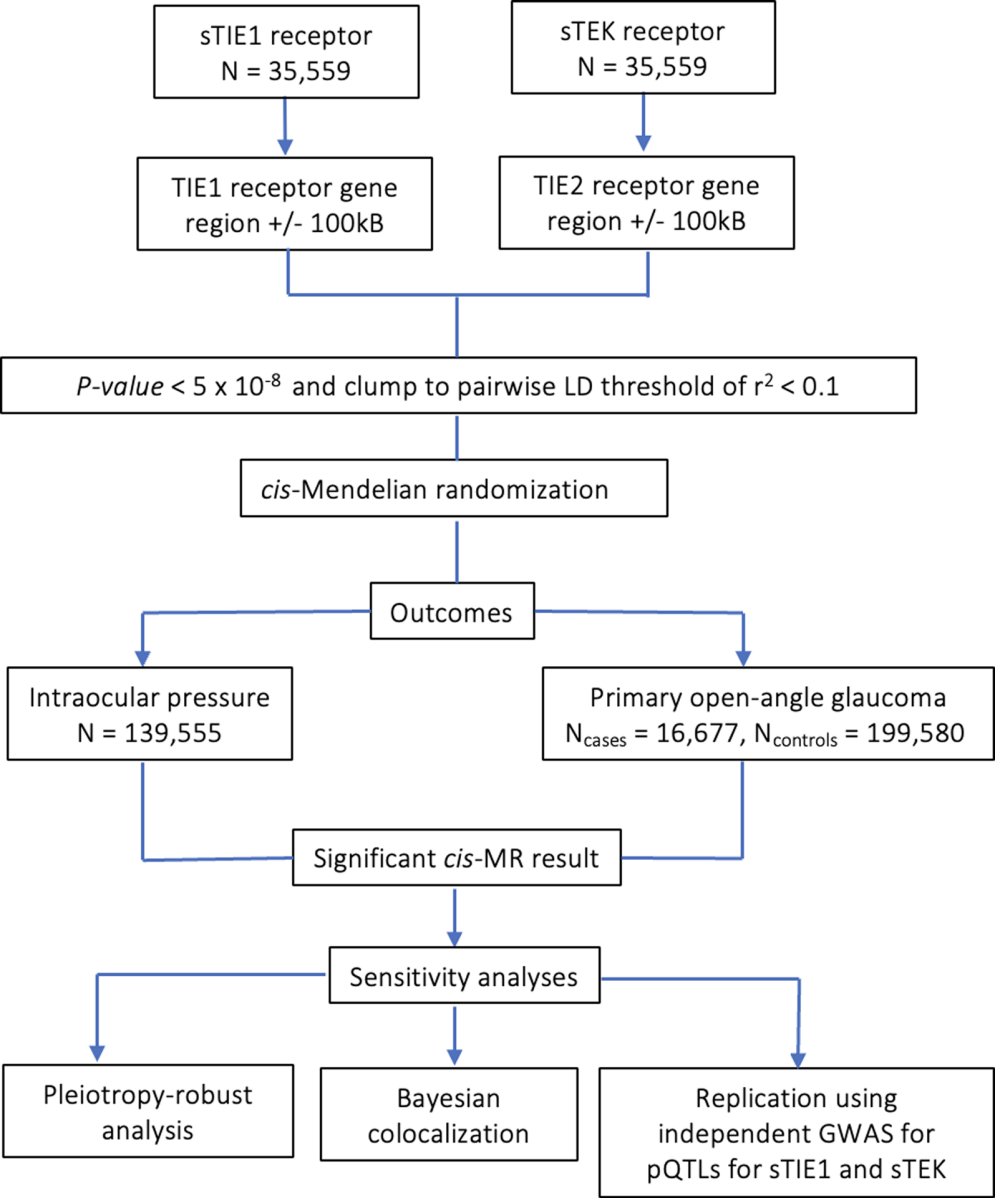
Flowchart outlining the study design. Study design and statistical analysis plan. Gene coordinates for drug-target gene region obtained from *Ensembl* (version 108, genome build GRCh38). *MR* Mendelian Randomization, *GWAS* genome-wide association study, *LD* linkage disequilibrium

A flowchart illustrating the statistical analysis plan is shown in Fig. 1. First, two-sample *cis-*Mendelian randomization was performed to investigate associations of increased genetically proxied TIE1 and TEK signalling with IOP and liability to POAG. Where a significant MR association was identified, Bayesian colocalization analysis was performed to assess the likelihood that this MR estimate was attributable to genetic confounding through a variant in linkage disequilibrium (LD) with the genetic instrument. Finally, we investigated differential cellular mRNA expression of *TIE1* and *TEK* in the human ocular anterior segment by analysing publicly available single-nucleus transcriptomic data.

### Data sources

We leveraged GWAS data in European ancestry individuals for all exposures and outcomes. GWAS data for circulating protein levels of sTIE1 and sTEK and corresponding pQTL data were obtained from the deCODE Genetics GWAS of the plasma proteome of 35,559 Icelandic individuals [24]. For IOP, summary statistics were obtained from the largest GWAS of IOP performed to date (*N* = 139,555), combining data from the UK Biobank (UKBB), EPIC-Norfolk, and the International Glaucoma Genetics Consortium (IGGC) [12]. For POAG, summary statistics were obtained from the largest GWAS meta-analysis of POAG performed in European ancestry individuals (*N*_*cases*_ = 16,677, *N*_*controls*_ = 199,580) [13]. A summary of these GWASs is provided in Additional file 1: Table S1. We also leveraged single-nucleus RNA-seq data in six tissues from the van Zyl et al*.* [25] cell atlas of the human ocular anterior segment. The study was reported in line with the ‘strengthening the reporting of observational studies in epidemiology using mendelian randomization’ (STROBE-MR) guidelines (Additional file 15: Table S15) [26].

### Genetic instruments

To proxy TIE1 and TEK signalling perturbation, we selected near-uncorrelated (pairwise LD threshold of *r*^*2*^ < 0.1) variants associated with circulating sTIE1 or sTEK protein levels at genome-wide significance (*P* < 5 × 10^–8^) located within the *TIE1* and *TEK* gene regions ± 100kB gene regions (Fig. 1). Publicly available genomic coordinates from *Ensembl* version 108 with reference to the appropriate reference genome panel for this GWAS (GRCh38/hg38) were used to identify the *TIE1* (chr1: 43,300,982–43,323,108) and *TEK* (chr9: 27,109,141–27,230,176) gene coordinates. The use of a ± 100 kB window reduces the risk of horizontal pleiotropy whilst still allowing variants outside the protein coding region that may regulate protein expression to be sampled. PLINK v2.0 [27] and phase 3, version 5 of the 1000 Genomes European reference panel [28] was used to perform LD clumping, which helps ensure that each instrumental variant represents independent biological signals, thus avoiding overestimation of instrument strength. Further details, including a table of the drug target gene, genomic coordinates, number of instrumental variants, R^2^ and F-statistics, are reported in Additional file 2: Table S2. The F-statistic quantifies the strength of the relationship between the genetic instrument and the exposure and a value > 10 indicates a strong instrument [29]. The R^2^ value quantifies the proportion of the variance in the exposure explained by the genetic instrument.

### Mendelian randomization

**Fig. 2:**
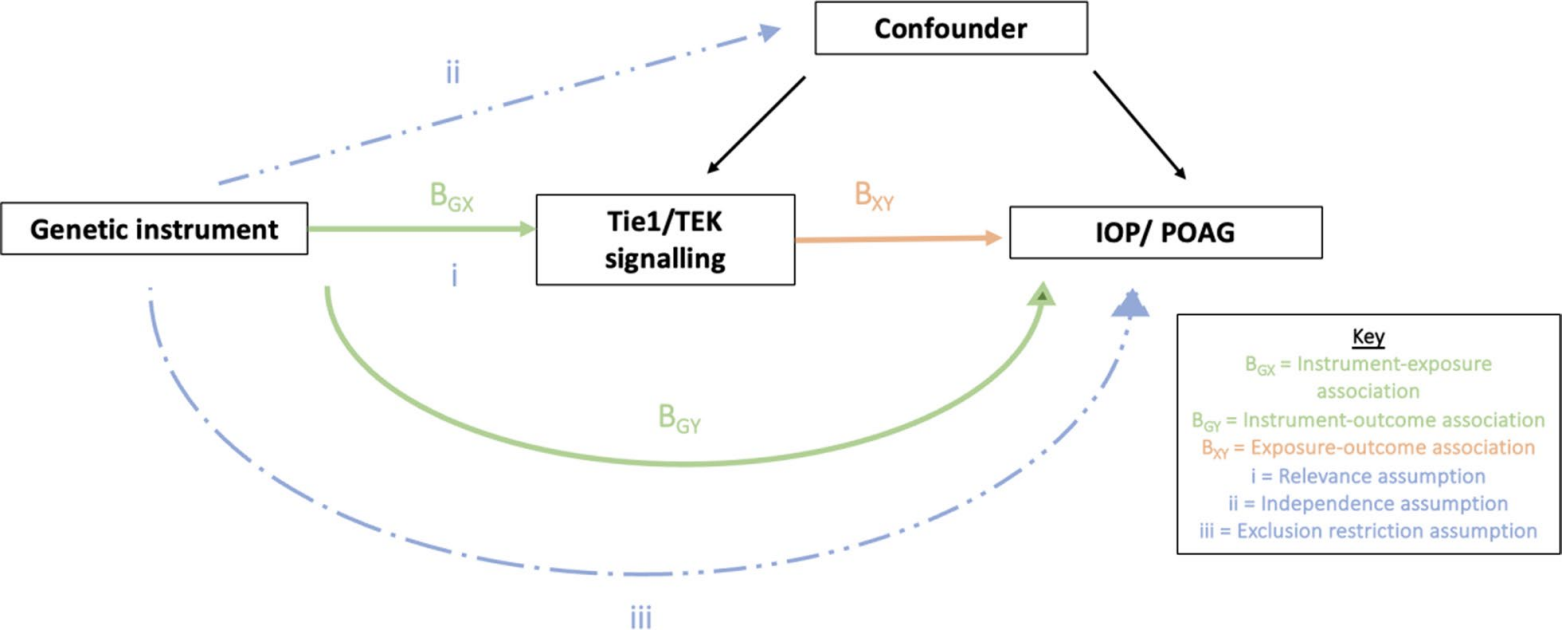
Direct Acyclic Graph illustrating core MR assumptions. The three core MR assumptions are: (i) the genetic instrument is robustly associated with the drug target, (ii) the genetic instrument is independent of any confounders, (iii) the genetic instrument influences IOP/POAG exclusively via the drug target (or factors downstream of the drug target). In the Wald ratio method, the instrument-outcome association (B_GY_) is divided by the instrument-exposure association (B_GX_). *IOP* intraocular pressure, *POAG* primary open-angle glaucoma. The broken lines represent forbidden pathways that constitute violations of the MR assumptions

Drug target MR relies on three core assumptions. First, the genetic instrument associates robustly with the drug target (relevance). Second, the genetic instrument shares no common cause with the outcome (independence). Third, the genetic instrument influences the outcome only via its effect on the drug target (exclusion-restriction) [30]. These are illustrated in Fig. 2. MR estimates for genetically proxied TIE1 signalling and TEK signalling were expressed per standard deviation (SD) increase in circulating sTIE1 and per SD decrease in circulating sTEK, respectively.

Genetic associations were harmonised by aligning effect alleles in both exposure and outcome datasets (Additional file 3: Table S3 and Additional file 4: Table S4). Using Ensembl’s variant effect predictor (VEP) annotation (https://genetics.opentargets.org/), information on how individual pQTLs may affect sTIE1 and sTEK levels based on genomic location are provided in Additional file 5: Table S5 and Additional file 6: Table S6. MR estimates were generated by calculating the Wald-ratio = the variant-outcome regression coefficient divided by the variant-exposure regression coefficient [31]. Where multiple variants comprised the instrument, the inverse-variance weighted (IVW) method was used to generate the MR effect estimate. The IVW approach assumes the absence of any horizontal pleiotropy, and so methods robust to the influence of pleiotropy and violation of the third MR assumption were used as sensitivity analyses [30]. The weighted-median [32], Contamination Mixture [33], MR-Egger [34] and MR-PRESSO [35] methods were applied. We also reported the *P*-values for the Egger-intercept test and the MR-PRESSO global heterogeneity test. Further details of these methods are provided in Additional file 23. Mendelian randomization analyses were performed using the *TwoSampleMR* [36] and *MendelianRandomization* [37] packages in R (version 4.1.2).

### Colocalization analysis

Coloc evaluates the likelihood of a shared vs distinct causal variants underlying the drug target gene region and outcome. Approximate Bayes Factors are calculated based on three prior probabilities: *p*1, *p*2 and *p*12, which represent the prior probabilities that any given variant in the drug target gene region (*TIE1* and *TEK*) is associated with either trait 1 (circulating levels of sTIE1/ sTEK), trait 2 (IOP), or both traits, respectively [38]. No single value of *p*12 is appropriate for all datasets and so we used Giambartolomei et al*.*’s recommended prior of *p*12 = 1 × 10^–5^, as proposed in the original methods paper [38], and *p*12 = 5 × 10^–5^, which subsequent studies have shown represents a reasonable balance between false positives and false negatives [39].

A high posterior probability of a shared causal variant (PP_shared_) relative to the posterior probability of distinct causal variants (PP_distinct_) supports a common causal pathway underlying TIE1 or TEK signalling and IOP. In contrast, a higher PP_distinct_ relative to the PP_shared_ supports distinct causal pathway underlying TIE1 or TEK signalling and IOP, indicating potential confounding by LD in the corresponding *cis-*MR result. In confounding by LD, two variants in the drug target gene region of interest are in LD or correlated with one another, one associating with the exposure and the other associating with the outcome [40]. The probability of colocalization conditional on the presence of a causal variant can be calculated as PP_shared_/(PP_distinct_ + PP_shared_). Colocalization analyses were performed using the *coloc* package in R (version 4.1.2). Further details are provided in Additional file 23.

### Replication

We replicated our *cis-*MR and coloc analyses using an independent GWAS for both sTIE1 and sTEK protein levels, Sun et al*.*’s GWAS of the human plasma proteome [41], which consisted of 3,301 healthy participants from the UK INTERVAL study.

### Single cell transcriptomic and differential gene expression analysis

No protein or gene expression quantitative trait loci are currently available for human ocular anterior segment tissues and hence our *cis-*MR and colocalization analyses could not use tissue-specific genetic association data. We therefore inspected the expression of *TIE1* and *TEK* in the single-nucleus RNA-seq dataset of six tissues (central cornea, corneoscleral wedge, trabecular meshwork/Schlemm’s canal, iris, ciliary body, and lens) from the van Zyl et al*.* [5, 25] cell atlas of the human ocular anterior segment. We plotted gene expression values (log(TPK + 1)) for *TIE1* and *TEK* across the 39 cell types detected and examined in which cell types *TIE1* and *TEK* were most highly expressed, based on differential gene expression analysis across cell types performed in van Zyl et al*.* [25]. Only genes expressed in more than 25% of cells in any cell type and with a log_2_(fold-change) above 0.25 were included in the analysis.

## Results

### cis-Mendelian randomization

### TIE1, TEK and IOP

**Fig. 3:**
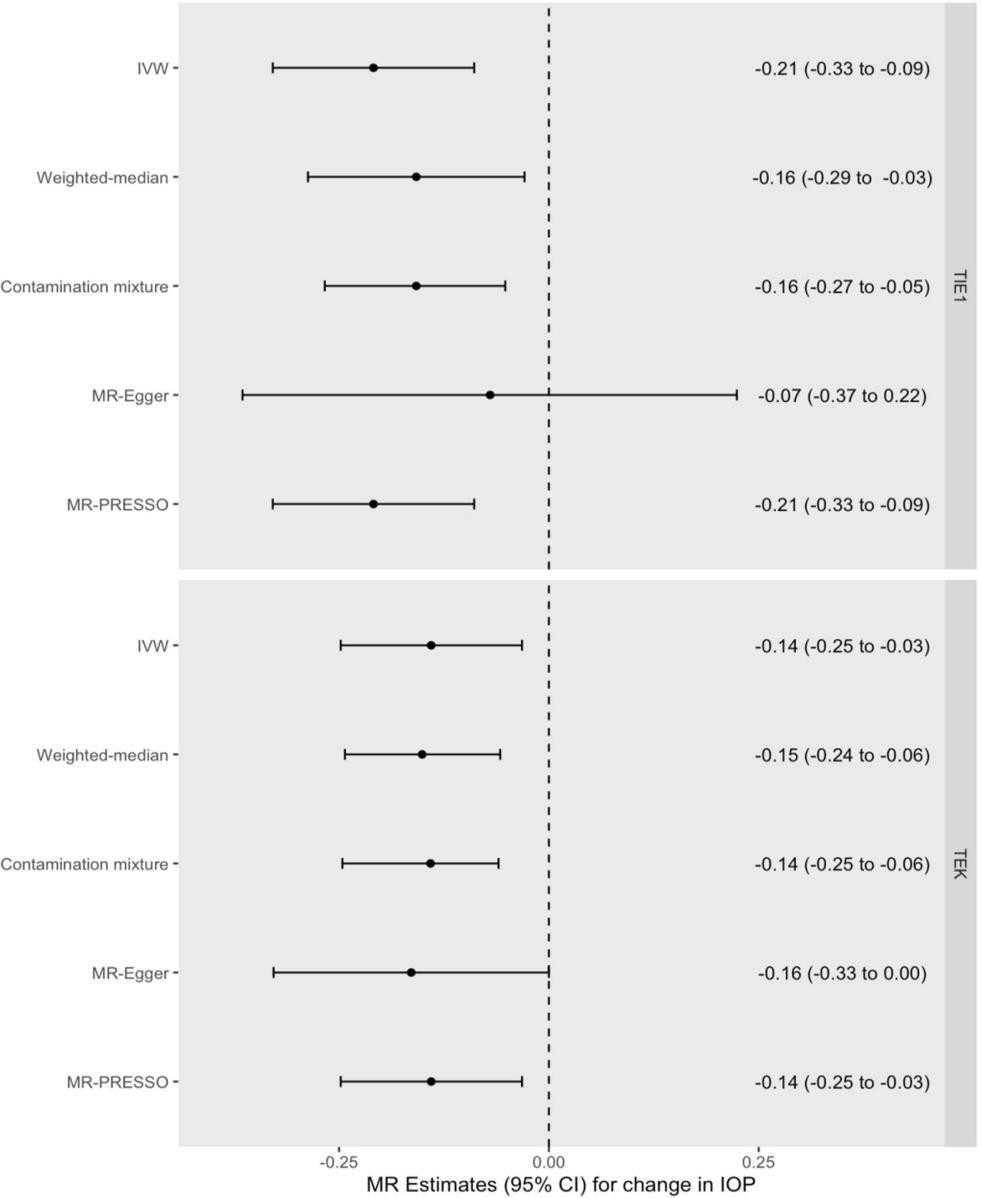
Forest plot of *cis-*MR estimates for increased TIE1 and TEK signalling on IOP. Forest plot illustrating MR estimates for the effect of increased genetically proxied TIE1 and TEK signalling on intraocular pressure, expressed as the mmHg change in IOP per SD increase in circulating sTIE1 and a SD decrease in circulating sTEK, respectively. Dot represents the point estimate and the lines the 95% confidence intervals. Dotted line represents line of null effect

Increased genetically proxied TIE1 signalling was associated with a 0.21 mmHg lower IOP per SD increase in circulating sTIE1 (95% CI = 0.09 to 0.33 mmHg, *P* = 6.57 × 10^–4^) (Fig. 3 and Additional file 7: Table S7). Increased genetically proxied TEK signalling was associated with a 0.14 mmHg lower IOP per SD decrease in circulating sTEK (95% CI = 0.03 to 0.25 mmHg, *P* = 0.011) (Fig. 3 and Additional file 8: Table S8). Results were consistent across pleiotropy-robust sensitivity analyses (Fig. 3) and were not driven by any one single variant (Additional file 16: Figure S1, Additional file 17: Figure S2, Additional file 18: Figure S3, Additional file 19: Figure S4). Replication using an independent GWAS for circulating sTIE1 and sTEK yielded similar results (Additional file 7: Tables S7 and Additional file 10: Table S10).

### TIE1, TEK and POAG

**Fig. 4:**
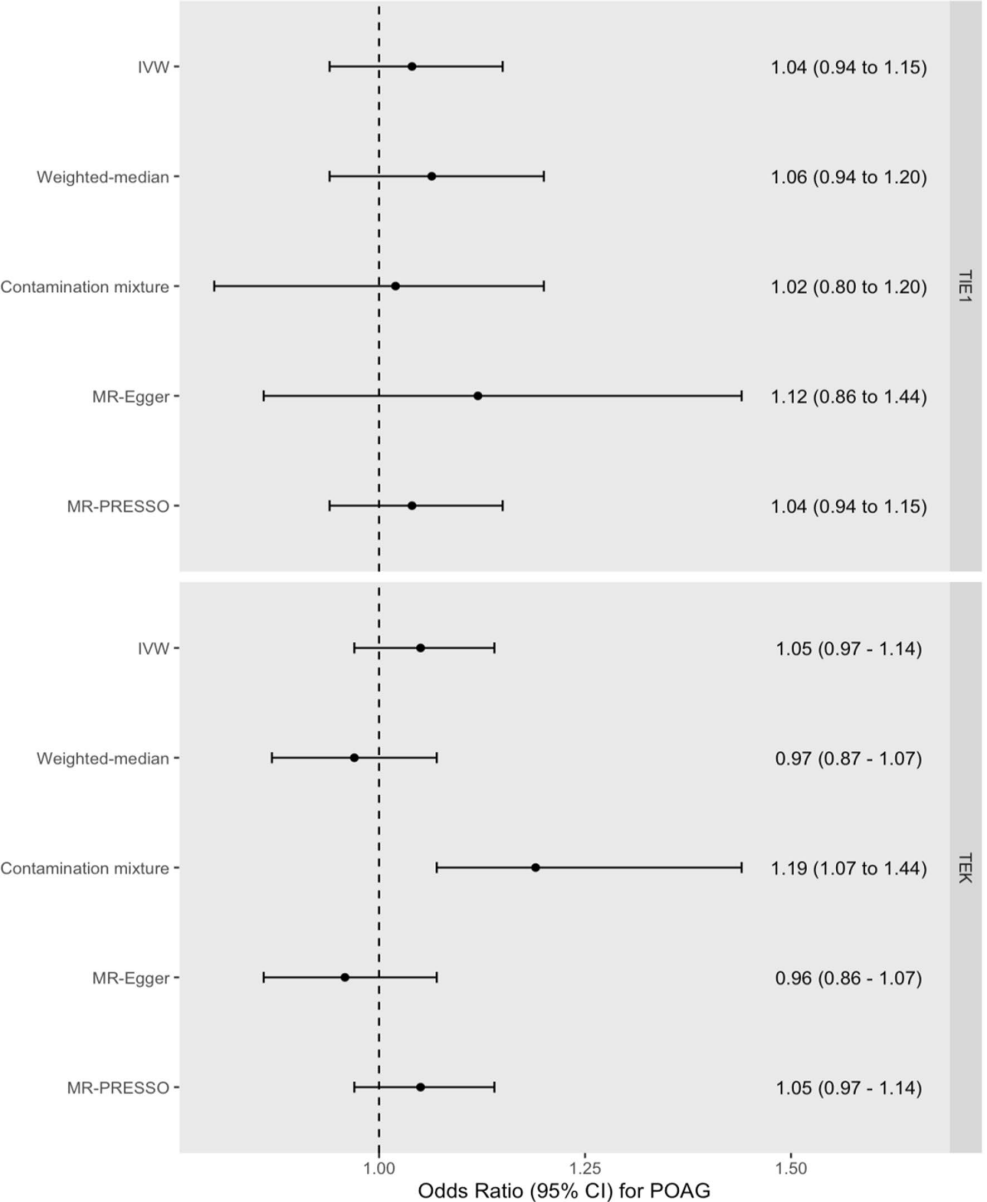
Forest plot of *cis-*MR estimates for increased TIE1 and TEK signalling on liability to POAG. Forest plot illustrating MR estimates for the effect of increased genetically proxied TIE1 and TEK signalling on liability to POAG, expressed as the odds ratio (OR) for POAG per SD increase in circulating sTIE1 and a SD decrease in circulating sTEK, respectively. Dot represents the point estimate and the lines the 95% confidence intervals. Dotted line represents line of null effect

We did not identify an association between increased genetically proxied TIE1 signalling and liability to POAG (OR = 1.04 per SD increase in circulating sTIE1, 95% CI = 0.94 to 1.15, *P* = 0.479) (Fig. 4 and Additional file 7: Table S7). Results were consistent across pleiotropy-robust sensitivity analyses for TIE1. Similarly, we did not identify an association between increased genetically proxied TEK signalling and liability to POAG (OR = 1.05 per SD decrease in circulating sTEK, 95% CI = 0.97 to 1.14, *P* = 0.243) (Fig. 4 and Additional file 8: Table S8). However, results were inconsistent across pleiotropy-robust sensitivity analyses (Fig. 4) for TEK, and the Egger intercept test indicated the presence of horizontal pleiotropy (*P* = 0.042).

### Colocalization analysis

### TIE1 and IOP

**Fig. 5:**
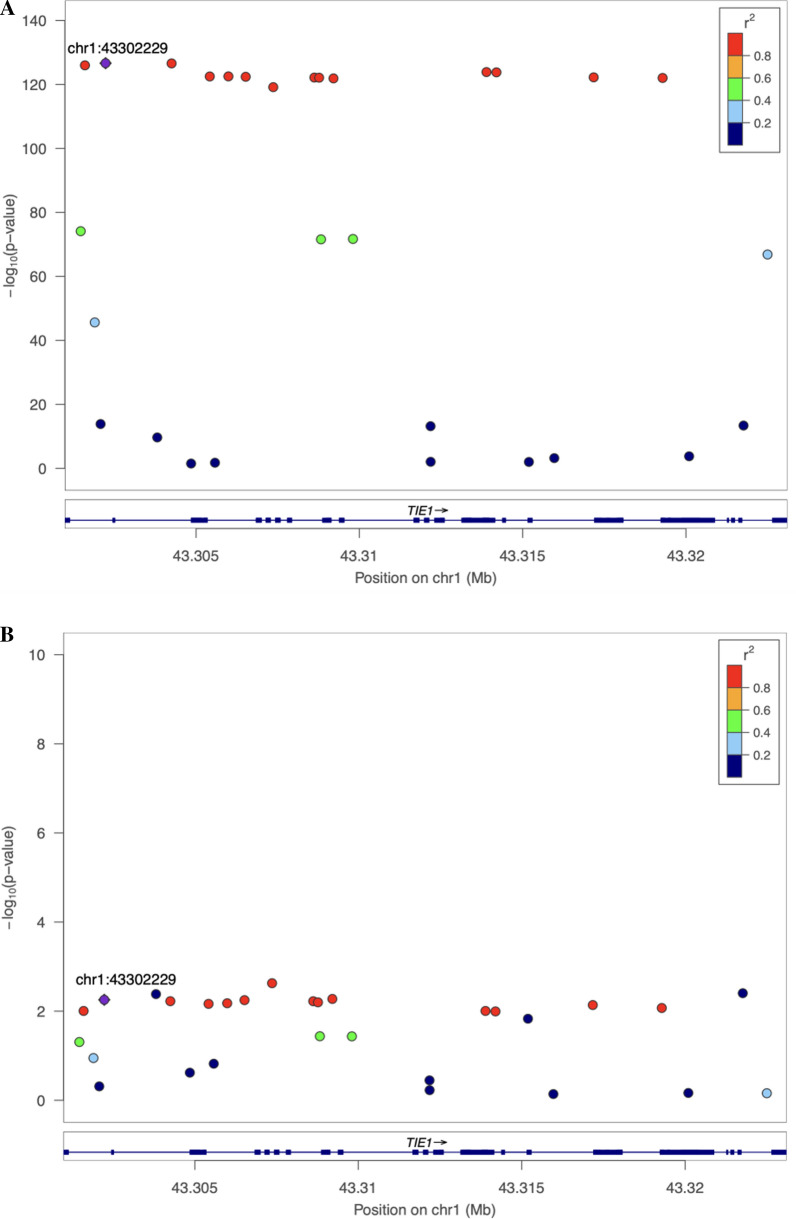
LocusZoom plot for sTIE1 protein levels and IOP in *TIE1* gene region. LocusZoom plot of genetic associations of variants within *TIE1* gene region with circulating sTIE1 protein levels (**A**) and IOP (**B**). The purple diamond is the variant with the highest posterior probability of being the shared causal variant underlying the two traits (PP_shared_), as determined by coloc. Points are color-coded based on their LD (r^2^) relative to the variant with the highest PP_shared_. LocusZoom plot of sTIE1 genetic associations using a 100kB window is shown in Additional file 22: Figure S7

**Fig. 6:**
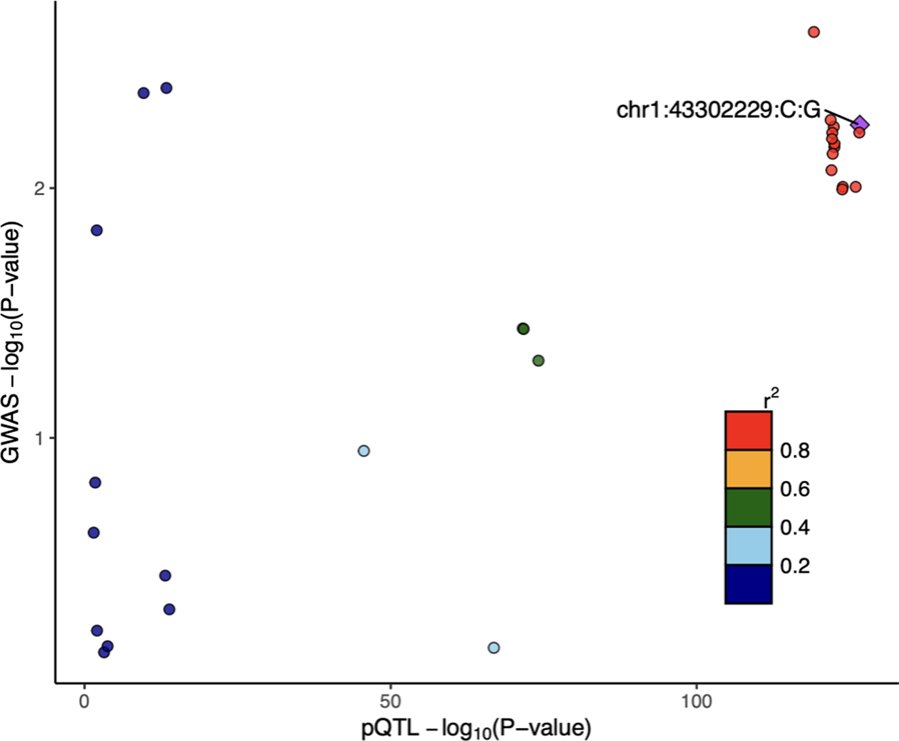
LocusCompare plot for sTIE1 protein levels and IOP in the *TIE1* gene region. LocusCompare plot illustrating genetic associations within *TIE1* gene region between circulating sTIE1 protein levels (x-axis) and IOP (y-axis). Each data point represents a genetic variant. The purple diamond is the genetic variant with the strongest association with sTIE1 protein levels. Points are color-coded based on each variant’s LD (r^2^) relative to the variant with the highest colocalization posterior probability in the gene region

Colocalization analysis supported the presence of a shared causal variant underlying TIE1 receptor signalling and IOP (Table 1 and Additional file 11: Table S11). At both prior probabilities of p12 = 1 × 10^–5^ and p12 = 5 × 10^–5^, the posterior probability of sTIE1 and IOP sharing a causal variant, PP_shared_, was orders of magnitude greater than the posterior probability of distinct causal variants underlying sTIE1 and IOP, PP_distinct_, at 53- and 262-fold greater, respectively. The posterior probability of a shared causal variant, conditional on the presence of one or more causal variants, PP_shared_ / (PP_distinct_ + PP_shared_), was 0.98 in both cases. Coloc findings were replicated using an independent GWAS for circulating sTIE1 protein levels (Additional file 12: Table S12). Consistent with this, LocusZoom and LocusCompare plots in Figs. 5 and 6, respectively, show a concordant distribution of genetic associations in the *TIE1* gene region with both sTIE1 and IOP. Of the variants in the gene region, the variant with the highest PP_shared_ exhibits relatively strong associations with both sTIE1 and IOP, with highly correlated variants exhibiting similarly strong associations and less correlated variants exhibiting progressively weaker associations.

**Table 1:** Coloc results for TIE1 or TEK signalling and IOP

Drug Target	p12	PP_distinct_	PP_shared_	PP_shared_/(PP_distinct_ + PP_shared_)
TIE1 signalling	1 × 10^–5^	0.002	0.10	0.98
	5 × 10^–5^	0.001	0.37	0.98
TEK signalling	1 × 10^–5^	0.25	0.11	0.30
	5 × 10^–5^	0.18	0.37	0.68

Coloc results. PP_distinct_: Posterior probability of a distinct causal variant for TIE1/TEK and IOP (PP_distinct_); PP_shared_: Posterior probability of a shared causal variant for TIE1/TEK and IOP; PP_shared_ /(PP_distinct_ + PP_shared_**):** Posterior probability of a shared causal variant for TIE1/TEK and IOP conditional on the presence of one or more causal variants (PP_shared_/PP_shared_ + PP_distinct_). Results are reported for two prior probabilities of a shared causal variant for TIE1/TEK and IOP (p12): p12 = 1 × 10^–5^ and 5 × 10^–5^

### TEK and IOP

Colocalization analysis yielded mixed evidence in support of a shared causal variant underlying TEK receptor signalling and IOP (Table 1 and Additional file 11: Table S11). At p12 = 1 × 10^–5^, PP_distinct_ was approximately 2.5-fold that of PP_shared_; conversely, at p12 = 5 × 10^–5^, PP_shared_ was approximately twice that of PP_distinct_. At these two priors, PP_shared_/(PP_distinct_ + PP_shared_) was 0.30 and 0.68, respectively. Replication using an independent GWAS for circulating sTEK yielded similar results (Additional file 12: Table S12). Consistent with this, LocusZoom and LocusCompare plots do not show a concordance of genetic associations in the TEK gene region for sTEK and IOP (Additional file 20: Figure S5 and Additional file 21: Figure S6).

### Single cell expression in six tissues in the anterior segment of the eye

**Fig. 7:**
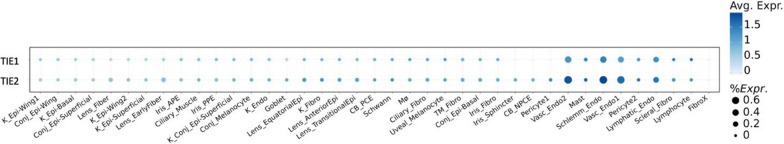
Single-nucleus RNA sequencing expression of *TIE1* and *TEK* in anterior segment of human ocular tissue. Dot plots illustrating single-nucleus RNA-sequencing expression levels of *TIE1* and *TEK* in the anterior segment of non-diseased human eyes. The size of each dot reflects the fraction of cells expressing mRNA for each gene. The color intensity depicts the average normalized gene expression levels (log(TPK + 1)) in expressing cells for the given cell type. A full list of abbreviations is provided in Additional file 14: Table S14

Upon inspection of *TIE1* and *TEK* single nucleus RNA-sequencing expression levels and differential gene expression across 39 cell types in six tissues in the ocular anterior segment (cornea, corneoscleral wedge, trabecular meshwork/Schlemm’s canal, ciliary body, iris, and lens) [25], we found that *TIE1* and *TEK* are preferentially and most highly expressed in SC endothelium, vascular endothelium, and lymphatic endothelium cells (*P* < 1 × 10^–53^ for associations) (Fig. 7 and Additional file 13: Table S13).

## Discussion

We performed *cis-*MR and colocalization to investigate the causal effect of increased TIE1 and TEK receptor signalling on IOP and liability to POAG. We find novel, human-centric genetic evidence to support a causal effect of increased TIE1/TEK signalling in lowering IOP, with stronger support for TIE1 than TEK.

### TIE1 signalling and IOP

We found that increased genetically proxied TIE1 signalling associated with lower IOP. Recent evidence shows that knocking out the full-length TIE1 receptor in mice results in abnormal SC development and raised IOP [3], pointing to an indispensable role for TIE1 signalling in IOP regulation. TIE1 is an orphan receptor that does not directly bind ANGPT1 or ANGPT2 but interacts with TEK via heterodimerization [8]. Molecular studies investigating the interaction between TIE1 and TEK have shown that ectodomain shedding and the release of sTIE1 facilitates ANGPT1-mediated activation of membrane-bound TEK receptors [7, 8]. The significant MR associations and aligned single cell expression patterns reported here for both TIE1 and TEK signalling are consistent with the idea that both receptors act cooperatively to regulate IOP in humans, thereby providing further clarity on the role of TIE1 receptors in relation to that of TEK receptors. Moreover, our *cis-*MR results were supported by colocalization at both conservative (p12 = 10^–5^) and liberal priors (p12 = 5 × 10^–5^), suggesting that our TIE1 MR result is unlikely to be driven by confounding by LD. Even at the more conservative prior, there was a 52-fold higher probability of a shared causal variant as opposed to distinct causal variants underlying both TIE1 signalling and IOP. Thus, building on recent animal data [3], our results provide novel human genetic support for a causal effect of increased TIE1 signalling on IOP-lowering in humans. This is supported by emerging data implicating heterozygous TIE1 mutations in the development of childhood glaucoma [42]. Taken together, these findings raise the possibility that perturbation of TIE1 signalling represents a therapeutic target in addition to, or independently of, TEK receptor perturbation. It should be noted that the absence of a natural endogenous ligand for TIE1 need not preclude its perturbation pharmacologically. For instance, the recreational drug, phenylcyclohexyl piperidine (PCP), binds to an orphan receptor in the NMDA receptor to induce potent dissociative and hallucinogenic effects [43].

### TEK signalling and IOP

We found that increased genetically proxied TEK signalling associated with lower IOP, though to a lesser extent than TIE1 signalling. Rare mutations in the *TEK* gene that lead to loss of TEK protein function were shown to be present in a subset of families with primary congenital glaucoma (PGC) [9]. Previous animal studies suggest a protective role for TEK receptor signalling in regulating IOP and reducing liability to POAG phenotypes. For example, adult mice deficient in both *ANGPT1*/*ANGPT2* or *TEK* develop impaired SC integrity and retinal ganglion cell dysfunction [10, 44] that can be rescued by TEK reactivation [10]. In humans, previous large-scale GWAS data identified significant associations for variants in the *ANGPT1* and *ANGPT2* gene regions with IOP [12] and POAG [13], and eQTLs targeting *ANGPT1* and *ANGPT2* have recently been shown to colocalize with IOP [45]. However, no GWAS associations have previously been observed in the *TIE1* or *TEK* gene regions. Our *cis-*MR results provide novel human-centric genetic support for a protective effect of TEK receptor signalling in reducing IOP and consistent with this, single cell RNA expression data shows that *TEK* is preferentially expressed in SC, vascular and lymphatic endothelial cells in the human ocular anterior segment. However, genetic support from colocalization analysis was mixed, with a comparatively higher probability of a shared causal variant at *p*12 = 5 × 10^–5^, but a comparatively higher probability of distinct causal variants at *p*12 = 10^–5^, indicating potential confounding by LD in our TEK MR result. Interestingly, a recent phase II double-blind RCT in patients with ocular hypertension or POAG found that twice daily application of topical Razuprotafib, which activates TEK receptors via vascular endothelial protein tyrosine phosphatase (VE-PTP) inhibition, reduced IOP when given in addition to latanoprost [46]. The comparatively stronger genetic support for TIE1 perturbation identified in this study, raises the possibility that an IOP-lowering effect may additionally be achieved by drugs stimulating TIE1 receptors.

### TIE1, TEK signalling and POAG

In this study, we did not identify an association between genetically proxied TIE1 or TEK signalling with liability to POAG. One possible reason is insufficient power in the present study. The dichotomous nature of POAG outcome data and scope for phenotypic misclassification and heterogeneity amongst POAG cases in the underlying GWAS can reduce power to detect genetic associations with POAG liability. Furthermore, in *cis-*MR, variant selection is restricted to the vicinity of the drug target gene region and so fewer variants comprise the genetic instrument than in standard MR, where variants are sampled from throughout the entire genome. Whilst the inclusion of *trans* variants distal to the drug target gene region may increase power, it also significantly increases the risk of horizontal pleiotropy and in turn undermines causal inference [16, 19]. Considering the overall null result, one also cannot rule out the possibility that any beneficial effect of IOP reduction is concurrently opposed by as yet poorly understood deleterious effects of increased TIE1/TEK signalling.

### Strengths

This study has several strengths. Prior evidence for functional consequences of altered TIE1/TEK signalling is primarily derived from animal studies. Although such studies are invaluable in understanding the molecular mechanisms via which TIE1/TEK signalling may affect IOP and POAG liability, they exhibit a high false discovery rate—estimated as high as 92.6% [14]—in yielding efficacious drug targets in humans. Furthermore, conventional observational methods investigating the effect of TIE1/TEK signalling are vulnerable to residual confounding and reverse causation. MR leverages the randomly allocated and non-modifiable nature of genetic variants to reduce unmeasured confounding and reverse causation, and thus produce human-centric causal association estimates. Given that individuals are blinded to their genotype, the results are also robust to the influence of ascertainment bias [15]. A growing body of empirical evidence supports the integration of genetics into drug discovery efforts, with prior genetic evidence for a drug target significantly reducing the risk of late-stage failure in subsequent clinical trials in humans [17, 47]. Indeed, genetic associations with complex diseases often correspond to genomic regions enriched for known drug targets of FDA-approved medications for the given disease [48, 49].

The strength of causal inference with MR is contingent on the validity of the core MR assumptions. In this respect, the use of *cis-*pQTLs at stringent inclusion thresholds of *P* < 5 × 10^–8^ increases the likelihood that the instrumental variants functionally relate to TIE1 and TEK signalling, thereby strengthening the biological plausibility of the relevance assumption. With respect to the exclusion-restriction assumption, we leveraged variants within 100kB of TIE1 and TEK gene regions, thereby reducing the likelihood of direct effects of our genetic instrument on IOP or POAG liability not exclusively via TIE1/TEK signalling. The use of *cis-*pQTLs is also important because the impact of prior genetic support for drug target success is greatest when genetic variants from protein-coding regions of the genome are used [18]. Finally, we replicated our *cis-*MR results using an independent GWAS for sTIE1 and sTEK protein levels.

### Limitations

Our study has some limitations. We assume that plasma levels of sTIE1 and sTEK directly relate to the degree of membrane-bound TIE1/TEK receptor signalling. However, this relationship may be confounded by variability in the levels of ANGPT1 and ANGPT2, the levels of TIE1 and TEK receptor cleavage, and the duration that cleavage products remain in the plasma (as determined by the stability of the cleaved protein and processes removing them from the circulation). Here, we used TIE1/TEK receptor levels as surrogates for TIE1/TEK receptor signalling but future studies could explore perturbation of ANGPT1, the main ligand for TEK receptors. Furthermore, the molecular mechanisms surrounding TIE1 and TEK ectodomain shedding, TIE1/TEK heterodimerization, and their resultant effects on angiopoietin-TEK signalling are still being understood. Consequently, the stronger genetic signal for TIE1 observed here could in part reflect sTIE1 levels being more representative of membrane-bound TIE1 signalling than sTEK levels are of membrane-bound TEK signalling. There is also uncertainty surrounding the extent to which TIE1 perturbation can be modelled as distinct from TEK perturbation, through the use of soluble TIE1 and TEK or otherwise.

MR estimates represent small lifelong genetic differences in TIE1/TEK signalling and so the magnitude of MR estimates are not interpretable on the same scale as those of a discrete clinical intervention, e.g., estimates derived from an RCT investigating TIE1/TEK signalling perturbation. Genetic data for exposures and outcomes were obtained exclusively from individuals of European ancestry to avoid confounding by ancestry and so the extent to which these findings can be generalised to other ancestries remains uncertain. The high combined values for PP_H0_, PP_H1_ and PP_H2_ (see Additional file 9: Table S9 and Additional file 10: Table S10) suggest our coloc analyses may be underpowered and so replication of these analyses once larger GWASs for TIE1, TEK, and IOP are available, is warranted. Nevertheless, replication of colocalization results using two independent plasma proteome GWASs suggests that these results are robust. It should also be noted that the *cis-*MR and colocalization analyses performed here are not specific to ocular tissue. Neither pQTL nor eQTL data are currently available for human ocular anterior segment tissue. Preferential *TIE1* and *TEK* mRNA expression in cells from aqueous humour outflow pathways in human ocular tissue suggests that it is biologically plausible that the genetic associations for IOP observed here reflect TIE1/TEK signalling in the anterior segment. Nevertheless, our study assumes that pQTLs in the *TIE1* and *TEK* gene regions affect SC protein levels as well systemic (plasma) protein levels, which further studies using pQTLs or eQTLs from ocular anterior segment tissue will be required to confirm.

## Conclusion

In this study, we find novel human genetic support for a causal role of both TIE1 and TEK signalling in regulating IOP. Furthermore, combined evidence from Mendelian randomization and colocalization analyses provide stronger support for TIE1 than TEK as a potential IOP-lowering therapeutic target. Further clinical studies investigating this prospect are warranted.

### Supplementary Information

**Additional file 1: Table S1.** Summary of Data Sources.

**Additional file 2: Table S2.** Drug target gene regions, number of instrumental variants, R^2^ and F-statistics.

**Additional file 3: Table S3.** Table of Mendelian randomization instrumental variants for TIE1 signalling perturbation.

**Additional file 4: Table S4.** Table of Mendelian randomization instrumental variants for TEK signalling perturbation.

**Additional file 5: Table S5.** Table of genomic location of instrumental variants for TIE1 pQTLs.

**Additional file 6: Table S6.** Table of genomic location of instrumental variants for TEK pQTLs.

**Additional file 7: Table S7.** Mendelian randomization estimates for the effect of increased genetically predicted TIE1 signalling (using deCODE Genetics GWAS of plasma proteome (N = 35,559)) on IOP and POAG.

**Additional file 8: Table S8.** Mendelian randomization estimates for the effect of increased genetically predicted TEK signalling (using deCODE Genetics GWAS of plasma proteome (N = 35,559)) on IOP and POAG.

**Additional file 9: Table S9.** Mendelian randomization estimates for the effect of increased genetically predicted TIE1 signalling (using Sun et al*.* [41] GWAS of plasma proteome (N = 3,301)) on IOP and POAG.

**Additional file 10: Table S10.** Mendelian randomization estimates for the effect of increased genetically predicted TEK signalling (using Sun et al*.* [41] GWAS of plasma proteome (N = 3,301)) on IOP and POAG.

**Additional file 11: Table S11.** Coloc analysis for Tie1, TEK and IOP using deCODE GWAS of plasma proteome (N = 35,559).

**Additional file 12: Table S12.** Colocalization analysis for Tie1, TEK and IOP using Sun et al. [41] GWAS of plasma proteome (N = 3,301).

**Additional file 13: Table S13.** Cell types with significant cell type specific expression for TIE1 and TIE2 in single-nucleus RNA sequencing data of the anterior segment of health human ocular tissues.

**Additional file 14: Table S14.** List of cell types identified in six anterior segment tissues from healthy human eyes based on clustering of single-nucleus RNA-sequencing data.

**Additional file 15: Table S15.** STROBE-MR Checklist.

**Additional file 16: Figure S1.** Leave-one-out plot for MR estimate of genetically proxied TIE1 and IOP.

**Additional file 17: Figure S2.** Leave-one-out plot for MR estimate of genetically proxied TIE1 and IOP.

**Additional file 18: Figure S3.** Scatter plot of the genetic associations of instrumental variants with sTIE1 and intraocular pressure.

**Additional file 19: Figure S4.** Scatter plot of the genetic associations of instrumental variants with sTEK and intraocular pressure.

**Additional file 20: Figure S5.** LocusZoom plot of genetic associations with sTEK protein levels and IOP in *TEK* gene region.

**Additional file 21: Figure S6.** LocusCompare plot of genetic associations with sTEK protein levels and IOP in the *TEK* gene region.

**Additional file 22: Figure S7.** LocusZoom plot of genetic associations with sTIE1 protein levels in the *TIE1* gene region with 100kB window.

**Additional file 23: Methods S1.** Supplementary Methods.

## Data Availability

All data sources used in this study are reported in Additional file 1: Table S1. POAG summary data can be downloaded at http://ftp.ebi.ac.uk/pub/databases/gwas/summary_statistics/GCST90011001-GCST90012000/GCST90011766/. sTIE1 and sTEK summary data can be downloaded at https://www.decode.com/summarydata/. IOP summary data may be available on reasonable request.
