# Supplementary material for: TIE1 and TEK signalling, intraocular pressure, and primary open-angle glaucoma: a Mendelian randomization study
Source: J Transl Med. 2023 Nov 24;21:847. doi: 10.1186/s12967-023-04737-9 (PMC10668387; doi:10.1186/s12967-023-04737-9)
Supplement: Supplementary file 1 — Additional file 1: Table S1. Summary of Data Sources. [file 12967_2023_4737_MOESM1_ESM.docx]

**Table S1 – Summary of data sources used in the present study**

| **Variable** | **Study** | **Ancestry** | **N individuals or N cases / controls** | **Case definition and ascertainment** | **PMID** |
| --- | --- | --- | --- | --- | --- |
| TIE1/ TEK signalling | Ferkingstad *et al.* 2021 [1] | EUR | 35,559 | Rank-based inverse normalised serum levels of circulating sTIE1 and sTEK adjusted for age, sex, and sample age.  Plasma protein levels were measured with the SomaScan version 4 assay (SomaLogic), which contains aptamers providing measurement of relative binding of the plasma sample to each of the aptamers in relative fluorescence units. | **34857953** |
| Intraocular pressure (IOP) | Khawaja *et al.* 2018 [2] | EUR | 139,555 | UK Biobank: IOP was measured once in each eye using the Ocular Response Analyzer (ORA, Reichert Inc.), a non-contact tonometer that measures the pressure on corneal flattening on both inward and outward motions, in response to a jet of air. These pressures were used to derive a Goldmann-correlated IOP (IOPg) and then corneal-compensated IOP (IOPcc), a measure of IOP least affected by corneal biomechanical properties.  EPIC Norfolk Eye Study: IOPcc was calculated as above for >94% participants.  IGGC: Goldmann applanation tonometry was the most common method of IOP measurement but approaches varied across the different individual studies. IOP was not corneal-corrected in all participating studies. Details can be found in the Supplementary Methods in Springelkamp *et al.* 2017.  For individuals reporting usage of IOP-lowering medication, measured IOP was divided by 0.7 based on average IOP reduction achieved with medication. | **29785010** |
| Primary open-angle glaucoma (POAG) | Gharahkhani *et al.* 2021 [3] | EUR | 16,677/ 199,580 | For most studies, POAG was defined according to International Classification of Diseases (ICD)-9 and ICD-10 criteria. Full details of case definitions can be found in the Supplementary Information of Gharahkhani *et al.* 2021. This study is the European subset of the cross-ancestry POAG meta-analysis. | **33627673** |

Table S1 – EUR = European. IGCC = International Glaucoma Genetics Consortium. PMID = PubMed Identification
