# Supplementary material for: TIE1 and TEK signalling, intraocular pressure, and primary open-angle glaucoma: a Mendelian randomization study
Source: J Transl Med. 2023 Nov 24;21:847. doi: 10.1186/s12967-023-04737-9 (PMC10668387; doi:10.1186/s12967-023-04737-9)
Supplement: Supplementary file 2 — Additional file 2: Table S2. Drug target gene regions, number of instrumental variants, R2 and F-statistics. [file 12967_2023_4737_MOESM2_ESM.docx]

**Table S2** – **Drug target gene regions, number of instrumental variants, R^2^ and F-statistics.**

| **Drug Target** | **Gene** | **Gene coordinates (GRCh38 / hg38)** | **Number of SNPs in genetic instrument** | **R^2^** | **F-statistic** |
| --- | --- | --- | --- | --- | --- |
| TIE1 receptor | *TIE1* | Chr1:43,300,982-43,323,108 | 9 | 0.0016 | 57.3 |
| TEK receptor | *TEK* | Chr9:27,109,141-27,230,178 | 12 | 0.0048 | 169 |

Table S2 - For TIE1 and TEK, instruments were generated from pQTL data using a P-value threshold < 5x 10^-8^ and a pairwise LD clumping threshold of r^2^< 0.1. Gene coordinates obtained from *Ensembl version 108*. Reported gene coordinates do not include +/- 100kB windows. The F-statistic was approximated using the formula F = ((Beta_exposure_/(SE_exposure_)^2^) and R^2^ approximated using the formula R^2^ = F/(N-2+F). *N* represents number of individuals in the GWAS. The F-statistic quantifies the strength of the relationship between the genetic instrument and the exposure. The R^2^ value quantifies the proportion of the variance in the exposure explained by the genetic instrument.
