# Supplementary material for: TIE1 and TEK signalling, intraocular pressure, and primary open-angle glaucoma: a Mendelian randomization study
Source: J Transl Med. 2023 Nov 24;21:847. doi: 10.1186/s12967-023-04737-9 (PMC10668387; doi:10.1186/s12967-023-04737-9)
Supplement: Supplementary file 3 — Additional file 3: Table S3. Table of Mendelian randomization instrumental variants for TIE1 signalling perturbation. [file 12967_2023_4737_MOESM3_ESM.docx]

**Table S3 – Table of Mendelian randomization instrumental variants for TIE1 signalling perturbation**

| Chromosome | Position | SNP | Effect Allele | Other Allele | Effect Allele Frequency | Beta (exposure) | Standard Error (exposure) | P-value (exposure) | Beta (outcome) | Standard Error (outcome) | P-value (outcome) |
| --- | --- | --- | --- | --- | --- | --- | --- | --- | --- | --- | --- |
| 1 | 43332563 | rs11576614 | T | g | 0.08018 | -0.1361 | 0.014779 | 3.29E-20 | 0.0873 | 0.0279 | 0.001747 |
| 1 | 43223072 | rs11585059 | A | g | 0.26214 | -0.0623 | 0.009088 | 7.14E-12 | 0.0123 | 0.015 | 0.4132 |
| 1 | 43302076 | rs1198979 | A | g | 0.12476 | 0.0939 | 0.012204 | 1.42E-14 | 0.0131 | 0.0189 | 0.487 |
| 1 | 43230037 | rs607949 | A | g | 0.13447 | -0.0846 | 0.011775 | 6.73E-13 | 0.0148 | 0.0208 | 0.477 |
| 1 | 43399295 | rs72671118 | T | c | 0.0876 | -0.1574 | 0.01412 | 7.35E-29 | 0.0401 | 0.0224 | 0.07385 |
| 1 | 43294565 | rs7549876 | T | g | 0.39975 | -0.1964 | 0.008119 | 2.91E-129 | 0.0297 | 0.0138 | 0.0314 |
| 1 | 43378597 | rs75575769 | A | c | 0.03599 | -0.1268 | 0.021444 | 3.36E-09 | -0.0105 | 0.0416 | 0.8001 |
| 1 | 43303811 | rs76148363 | T | g | 0.19967 | 0.0636 | 0.010028 | 2.27E-10 | -0.0547 | 0.0191 | 0.00414 |
| 1 | 43271271 | rs79825075 | T | c | 0.08021 | 0.1326 | 0.014615 | 1.16E-19 | 0.0029 | 0.0302 | 0.9247 |

exposure GWAS = sTIE1 levels

outcome GWAS = IOP
