# Supplementary material for: TIE1 and TEK signalling, intraocular pressure, and primary open-angle glaucoma: a Mendelian randomization study
Source: J Transl Med. 2023 Nov 24;21:847. doi: 10.1186/s12967-023-04737-9 (PMC10668387; doi:10.1186/s12967-023-04737-9)
Supplement: Supplementary file 4 — Additional file 4: Table S4. Table of Mendelian randomization instrumental variants for TEK signalling perturbation. [file 12967_2023_4737_MOESM4_ESM.docx]

**Table S4 – Table of Mendelian randomization instrumental variants for TEK signalling perturbation**

| Chromosome | Position | SNP | Effect Allele | Other Allele | Effect Allele Frequency | Beta (exposure) | Standard Error (exposure) | P-value (outcome) | Beta (outcome) | Standard Error (outcome) | P-value (outcome) |
| --- | --- | --- | --- | --- | --- | --- | --- | --- | --- | --- | --- |
| 9 | 27264541 | rs10967828 | T | g | 0.32156 | 0.0598 | 0.008803 | 1.10E-11 | 0.0066 | 0.0134 | 0.6233 |
| 9 | 27258312 | rs117741650 | A | g | 0.02451 | 0.1684 | 0.026929 | 4.01E-10 | 0.0192 | 0.038 | 0.6141 |
| 9 | 27126417 | rs1334809 | T | g | 0.17892 | 0.1167 | 0.010722 | 1.37E-27 | 0.0263 | 0.0194 | 0.1752 |
| 9 | 27283896 | rs139383988 | A | c | 0.0305 | 0.1428 | 0.024444 | 5.16E-09 | 0.1125 | 0.0391 | 0.004056 |
| 9 | 27041092 | rs148756899 | T | c | 0.02046 | 0.1882 | 0.028875 | 7.14E-11 | -1.00E-04 | 0.0499 | 0.9991 |
| 9 | 27133323 | rs17694761 | A | g | 0.3926 | -0.0569 | 0.008451 | 1.67E-11 | 0.0031 | 0.0132 | 0.814 |
| 9 | 27209471 | rs2273720 | A | c | 0.02097 | 0.5295 | 0.028935 | 8.35E-75 | -0.0731 | 0.0299 | 0.0145 |
| 9 | 27068921 | rs636330 | A | g | 0.14553 | 0.1257 | 0.011643 | 3.59E-27 | 0.0113 | 0.0218 | 0.6038 |
| 9 | 27200268 | rs671353 | A | g | 0.45487 | -0.0706 | 0.008252 | 1.17E-17 | 0.0344 | 0.0129 | 0.007477 |
| 9 | 27183465 | rs682632 | A | c | 0.05971 | -0.3853 | 0.017297 | 6.30E-110 | 0.0827 | 0.0326 | 0.01114 |
| 9 | 27187924 | rs75291040 | T | g | 0.05322 | -0.1082 | 0.018248 | 3.04E-09 | 0.0546 | 0.0263 | 0.0375 |
| 9 | 27170310 | rs994934 | T | c | 0.25218 | -0.0727 | 0.009498 | 1.94E-14 | 0.017 | 0.0152 | 0.2614 |

exposure GWAS = TEK levels

outcome GWAS = IOP
