# Supplementary material for: TIE1 and TEK signalling, intraocular pressure, and primary open-angle glaucoma: a Mendelian randomization study
Source: J Transl Med. 2023 Nov 24;21:847. doi: 10.1186/s12967-023-04737-9 (PMC10668387; doi:10.1186/s12967-023-04737-9)
Supplement: Supplementary file 5 — Additional file 5: Table S5. Table of genomic location of instrumental variants for TIE1 pQTLs. [file 12967_2023_4737_MOESM5_ESM.docx]

**Table S5 – Table of genomic location of instrumental variants for TIE1 pQTLs**

| Chromosome | Position | SNP | Effect Allele | Other Allele | Genomic Location |
| --- | --- | --- | --- | --- | --- |
| 1 | 43332563 | rs11576614 | T | g | Intergenic |
| 1 | 43223072 | rs11585059 | A | g | Intron |
| 1 | 43302076 | rs1198979 | A | g | Intron |
| 1 | 43230037 | rs607949 | A | g | Intron |
| 1 | 43399295 | rs72671118 | T | c | Intron |
| 1 | 43294565 | rs7549876 | T | g | Regulatory region variant |
| 1 | 43378597 | rs75575769 | A | c | Intergenic |
| 1 | 43303811 | rs76148363 | T | g | Intron |
| 1 | 43271271 | rs79825075 | T | c | Intron |

Genomic locations were identified using Ensemble’s variant effect predictor (VEP) (<https://genetics.opentargets.org/>). Intronic, intergenic and regulatory region variants likely affect protein expression levels through transcriptional and post-transcriptional gene regulation. However, the precise mechanisms through which many of the pQTLs affect sTIE1 protein levels are currently unknown.
