# Supplementary material for: TIE1 and TEK signalling, intraocular pressure, and primary open-angle glaucoma: a Mendelian randomization study
Source: J Transl Med. 2023 Nov 24;21:847. doi: 10.1186/s12967-023-04737-9 (PMC10668387; doi:10.1186/s12967-023-04737-9)
Supplement: Supplementary file 6 — Additional file 6: Table S6. Table of genomic location of instrumental variants for TEK pQTLs. [file 12967_2023_4737_MOESM6_ESM.docx]

**Table S6 – Table of genomic location of instrumental variants for TEK pQTLs**

| Chromosome | Position | SNP | Effect Allele | Other Allele | Genomic Location |
| --- | --- | --- | --- | --- | --- |
| 9 | 27264541 | rs10967828 | T | g | Intron |
| 9 | 27258312 | rs117741650 | A | g | Intron |
| 9 | 27126417 | rs1334809 | T | g | Intron |
| 9 | 27283896 | rs139383988 | A | c | Upstream gene variant |
| 9 | 27041092 | rs148756899 | T | c | Intron |
| 9 | 27133323 | rs17694761 | A | g | Intron |
| 9 | 27209471 | rs2273720 | A | c | Intron |
| 9 | 27068921 | rs636330 | A | g | Intergenic |
| 9 | 27200268 | rs671353 | A | g | Intron |
| 9 | 27183465 | rs682632 | A | c | Missense |
| 9 | 27187924 | rs75291040 | T | g | Intron |
| 9 | 27170310 | rs994934 | T | c | Intron |

Genomic locations were identified using Ensemble’s variant effect predictor (VEP) (<https://genetics.opentargets.org/>). Intronic, intergenic and upstream gene variants likely affect transcriptional and post-transcriptional gene regulation and thus protein expression levels whereas the missense variant likely affects protein stability. However, the precise mechanisms through which many of the pQTLs affect sTEK protein levels are currently unknown.
