# Supplementary material for: TIE1 and TEK signalling, intraocular pressure, and primary open-angle glaucoma: a Mendelian randomization study
Source: J Transl Med. 2023 Nov 24;21:847. doi: 10.1186/s12967-023-04737-9 (PMC10668387; doi:10.1186/s12967-023-04737-9)
Supplement: Supplementary file 7 — Additional file 7: Table S7. Mendelian randomization estimates for the effect of increased genetically predicted TIE1 signalling (using deCODE Genetics GWAS of plasma proteome (N = 35,559)) on IOP and POAG. [file 12967_2023_4737_MOESM7_ESM.docx]

**Table S7 - Mendelian randomization estimates for the effect of increased genetically predicted TIE1 signalling (using deCODE Genetics GWAS of plasma proteome (N = 35,559)) on IOP and POAG**

| P-value and LD clumping threshold | Outcome | No. of SNPs | MR Method | Beta/OR (95% CI) | P-value | MR-Egger intercept P-value | MR-PRESSO Global Heterogeneity Test P-value |
| --- | --- | --- | --- | --- | --- | --- | --- |
| P < 5e-8 & r^2^ < 0.1 | IOP | 9 | IVW | -0.209 (-0.33 to -0.09) | 6.57E-04 |  |  |
|  |  |  | ConMix | -0.158 (-0.267 to -0.052) | 0.008 |  |  |
|  |  |  | Weighted Median | -0.158 (-0.29 to -0.03) | 0.016 |  |  |
|  |  |  | MR-Egger | -0.070 (-0.36 to 0.22) | 0.64 | 0.312 |  |
|  |  |  | MR-PRESSO | -0.209 (-0.33 to -0.09) | 9.27E-03 |  | 0.288 |
| P < 5e-8 & r^2^ < 0.1 | POAG | 9 | IVW | 1.04 (0.94 to 1.15) | 0.48 |  |  |
|  |  |  | ConMix | 1.02 (0.80 to 1.20) | 0.65 |  |  |
|  |  |  | Weighted Median | 1.06 (0.94 to 1.20) | 0.35 |  |  |
|  |  |  | MR-Egger | 1.12 (0.86 to 1.44) | 0.40 | 0.540 |  |
|  |  |  | MR-PRESSO | 1.04 (0.94 to 1.15) | 0.50 |  | 0.519 |

MR effect estimates are scaled to a 1 standard deviation (SD) increase in sTIE1 circulating protein. IVW Beta (95% CI) is reported for IOP, and Odds Ratio (OR) (95% CI) is reported for POAG. IVW = inverse-variance weighted. CI = Confidence Interval. ConMix = Contamination Mixture.
