# Supplementary material for: TIE1 and TEK signalling, intraocular pressure, and primary open-angle glaucoma: a Mendelian randomization study
Source: J Transl Med. 2023 Nov 24;21:847. doi: 10.1186/s12967-023-04737-9 (PMC10668387; doi:10.1186/s12967-023-04737-9)
Supplement: Supplementary file 8 — Additional file 8: Table S8. Mendelian randomization estimates for the effect of increased genetically predicted TEK signalling (using deCODE Genetics GWAS of plasma proteome (N = 35,559)) on IOP and POAG. [file 12967_2023_4737_MOESM8_ESM.docx]

**Table S8 - Mendelian randomization estimates for the effect of increased genetically predicted TEK signalling (using deCODE Genetics GWAS of plasma proteome (N = 35,559)) on IOP and POAG**

| P-value and LD clumping threshold | Outcome | No. of SNPs | MR Method | Beta/OR (95% CI) | P-value | MR-Egger intercept P-value | MR-PRESSO Global Heterogeneity Test P-value |
| --- | --- | --- | --- | --- | --- | --- | --- |
| P < 5e-8 &  r^2^ < 0.1 | IOP | 12 | IVW | -0.140 (-0.25 to -0.03) | 0.011 |  |  |
|  |  |  | ConMix | -0.141 (-0.25 to -0.06) | 0.009 |  |  |
|  |  |  | Weighted Median | -0.151 (-0.24 to -0.06) | 0.001 |  |  |
|  |  |  | MR-Egger | -0.164 (-0.328 to 0.00) | 0.050 | 0.696 |  |
|  |  |  | MR-PRESSO | -0.140 (-0.25 to -0.03) | 0.027 |  | 0.057 |
| P < 5e-8 &  r^2^ < 0.1 | POAG | 14 | IVW | 1.05 (0.97 - 1.14) | 0.24 |  |  |
|  |  |  | ConMix | 1.19 (1.07 to 1.44) | 0.009 |  |  |
|  |  |  | Weighted Median | 0.97 (0.87 - 1.07) | 0.54 |  |  |
|  |  |  | MR-Egger | 0.96 (0.86 - 1.07) | 0.46 | 0.030 |  |
|  |  |  | MR-PRESSO | 1.05 (0.97 - 1.14) | 0.26 |  | 0.196 |

MR effect estimates are scaled to a 1 standard deviation (SD) decrease in sTEK circulating protein. IVW Beta (95% CI) is reported for IOP, and Odds Ratio (OR) (95% CI) is reported for POAG. IVW = inverse-variance weighted. CI = Confidence Interval. ConMix = Contamination Mixture.
