# Supplementary material for: TIE1 and TEK signalling, intraocular pressure, and primary open-angle glaucoma: a Mendelian randomization study
Source: J Transl Med. 2023 Nov 24;21:847. doi: 10.1186/s12967-023-04737-9 (PMC10668387; doi:10.1186/s12967-023-04737-9)
Supplement: Supplementary file 9 — Additional file 9: Table S9. Mendelian randomization estimates for the effect of increased genetically predicted TIE1 signalling (using Sun et al. [41] GWAS of plasma proteome (N = 3,301)) on IOP and POAG. [file 12967_2023_4737_MOESM9_ESM.docx]

**Table S9 - Mendelian randomization estimates for the effect of increased genetically predicted TIE1 signalling (using Sun *et al.* 2018 GWAS of plasma proteome (N = 3,301)) on IOP and POAG**

| P-value and LD clumping threshold | Outcome | No. of SNPs | MR Method | Beta/OR (95% CI) | P-value |
| --- | --- | --- | --- | --- | --- |
| P < 5e-8 &  r^2^ < 0.1 | IOP | 1 | Wald estimate | -0.16 (-0.26 to -0.06) | 0.002 |
| P < 5e-8 &  r^2^ < 0.1 | POAG | 1 | Wald estimate | 0.98 (0.89 to 1.09 | 0.75 |

MR effect estimates are scaled to a 1 standard deviation (SD) increase in sTIE1 circulating protein. Wald estimate beta (95% CI) is reported for IOP, and Odds Ratio (OR) (95% CI) is reported for POAG.
