# Supplementary material for: TIE1 and TEK signalling, intraocular pressure, and primary open-angle glaucoma: a Mendelian randomization study
Source: J Transl Med. 2023 Nov 24;21:847. doi: 10.1186/s12967-023-04737-9 (PMC10668387; doi:10.1186/s12967-023-04737-9)
Supplement: Supplementary file 11 — Additional file 11: Table S11. Coloc analysis for Tie1, TEK and IOP using deCODE GWAS of plasma proteome (N = 35,559). [file 12967_2023_4737_MOESM11_ESM.docx]

**Table S11 - Coloc analysis for Tie1, TEK and IOP using deCODE GWAS of plasma proteome (*N* = 35,559)**

| Tie1 | p12 | PP_H0_ | PP_H1_ | PP_H2_ | PP_H3_ | PP_H4_ | PP_H4_/PP_H3_ | PP_H4_/(PP_H3_+PP_H4_) |
| --- | --- | --- | --- | --- | --- | --- | --- | --- |
|  |  |  |  |  |  |  |  |  |
|  | p12 = 1e-5 | 1.21E-120 | 2.83E-123 | 0.894 | 0.002 | 0.104 | 52.5 | **0.98** |
|  |  |  |  |  |  |  |  |  |
|  | p12 = 5e-5 | 8.56E-121 | 2.00E-123 | 0.632 | 0.001 | 0.367 | 262.1 | **0.997** |
|  |  |  |  |  |  |  |  |  |
| TEK | **p12** | **PP_H0_** | **PP_H1_** | **PP_H2_** | **PP_H3_** | **PP_H4_** | **PP_H4_/PP_H3_** | **PP_H4_/(PP_H3_+PP_H4_)** |
|  |  |  |  |  |  |  |  |  |
|  | p12 = 1e-5 | 1.67E-102 | 6.46E-103 | 0.646 | 0.250 | 0.105 | 0.4 | **0.30** |
|  |  |  |  |  |  |  |  |  |
|  | p12 = 5e-5 | 1.18E-102 | 4.55E-103 | 0.455 | 0.176 | 0.369 | 2.1 | **0.68** |

p12 represents the prior probability of a shared causal variant underlying the drug target (Tie1 or TEK signalling) and IOP

PP_H0_ = posterior probability of there being no causal variant/association underlying the drug target or IOP

PP_H1_ = posterior probability that there is an association/causal variant underlying the drug target, but not IOP

PP_H2_ = posterior probability that there is an association/causal variant underlying IOP, but not the drug target

PP_H3_ = PPdistinct = posterior probability of distinct causal variants underlying the drug target and IOP

PP_H4_ = PPshared = posterior probability of a shared causal variant underlying the drug target and IOP

PP_H4_/(PP_H3_+PP_H4_) = the probability of a shared causal variant conditional on the presence of a causal variant (shared or distinct) underlying the drug target and IOP.

rs4660729 was the variant with the highest PPshared for TIE1 and rs682632 was the variant with the highest PPshared for TEK
