# Supplementary material for: TIE1 and TEK signalling, intraocular pressure, and primary open-angle glaucoma: a Mendelian randomization study
Source: J Transl Med. 2023 Nov 24;21:847. doi: 10.1186/s12967-023-04737-9 (PMC10668387; doi:10.1186/s12967-023-04737-9)
Supplement: Supplementary file 12 — Additional file 12: Table S12. Colocalization analysis for Tie1, TEK and IOP using Sun et al. [41] GWAS of plasma proteome (N = 3,301). [file 12967_2023_4737_MOESM12_ESM.docx]

| **Table S12 - Colocalization analysis for Tie1, TEK and IOP using Sun et al. 2018 GWAS of plasma proteome (N = 3,301)**   \| Tie1 \| p12 \| PP_H0_ \| PP_H1_ \| PP_H2_ \| PP_H3_ \| PP_H4_ \| PP_H4_/PP_H3_ \| PP_H4_/(PP_H3_+PP_H4_) \| \| --- \| --- \| --- \| --- \| --- \| --- \| --- \| --- \| --- \| \|  \|  \|  \|  \|  \|  \|  \|  \| \| p12 = 1e-5 \| 5.94E-18 \| 1.39E-20 \| 0.84 \| 0.002 \| 0.16 \| 88.9 \| 0.99 \| \|  \|  \|  \|  \|  \|  \|  \|  \| \| p12 = 5e-5 \| 3.62E-18 \| 8.46E-21 \| 0.51 \| 0.001 \| 0.49 \| 442.7 \| 0.997 \| \|  \|  \|  \|  \|  \|  \|  \|  \|  \| \| TEK \| p12 \| **PP_H0_** \| **PP_H1_** \| **PP_H2_** \| **PP_H3_** \| **PP_H4_** \| **PP_H4_/PP_H3_** \| **PP_H4_/(PP_H3_+PP_H4_)** \| \|  \|  \|  \|  \|  \|  \|  \|  \| \| p12 = 1e-5 \| 7.63E-17 \| 2.95E-17 \| 0.676 \| 0.261 \| 0.063 \| 0.2 \| 0.19 \| \|  \|  \|  \|  \|  \|  \|  \|  \| \| p12 = 5e-5 \| 6.11E-17 \| 2.36E-17 \| 0.541 \| 0.209 \| 0.250 \| 1.2 \| 0.54 \|   p12 represents the prior probability of a shared causal variant underlying the drug target (Tie1 or TEK signalling) and IOP  PP_H0_ = posterior probability of there being no causal variant/association underlying the drug target or IOP  PP_H1_ = posterior probability that there is an association/causal variant underlying the drug target, but not IOP  PP_H2_ = posterior probability that there is an association/causal variant underlying IOP, but not the drug target  PP_H3_ = PPdistinct = posterior probability of distinct causal variants underlying the drug target and IOP  PP_H4_ = PPshared = posterior probability of a shared causal variant underlying the drug target and IOP  PP_H4_/(PP_H3_+PP_H4_) = the probability of a shared causal variant conditional on the presence of a causal variant (shared or distinct) underlying the drug target and IOP. |
| --- | --- | --- | --- | --- | --- | --- | --- | --- | --- | --- | --- | --- | --- | --- | --- | --- | --- | --- | --- | --- | --- | --- | --- | --- | --- | --- | --- | --- | --- | --- | --- | --- | --- | --- | --- | --- | --- | --- | --- | --- | --- | --- | --- | --- | --- | --- | --- | --- | --- | --- | --- | --- | --- | --- | --- | --- | --- | --- | --- | --- | --- | --- | --- | --- | --- | --- | --- | --- | --- | --- | --- | --- | --- | --- | --- | --- | --- | --- | --- | --- | --- | --- | --- | --- | --- | --- | --- | --- | --- | --- | --- |
