# Supplementary material for: TIE1 and TEK signalling, intraocular pressure, and primary open-angle glaucoma: a Mendelian randomization study
Source: J Transl Med. 2023 Nov 24;21:847. doi: 10.1186/s12967-023-04737-9 (PMC10668387; doi:10.1186/s12967-023-04737-9)
Supplement: Supplementary file 13 — Additional file 13: Table S13. Cell types with significant cell type specific expression for TIE1 and TIE2 in single-nucleus RNA sequencing data of the anterior segment of health human ocular tissues. [file 12967_2023_4737_MOESM13_ESM.docx]

| **Table S13 - Cell types with significant cell type specific expression for TIE1 and TIE2 in single-nucleus RNA sequencing data of the anterior segment of health human ocular tissues.**   \| Gene \| Cell type \| Average gene expression, log(TPK+1) \| % cells in cell type expressing gene \| % cells in all other cell types expressing gene \| Average fold-change \| Adj. P-value* \| \| --- \| --- \| --- \| --- \| --- \| --- \| --- \| \| *TIE1* \| Schlemm_Endo \| 1.45 \| 0.424 \| 0.018 \| 2.48137125 \| 6.77E-143 \| \| *TIE1* \| Vasc_Endo1 \| 1.16 \| 0.325 \| 0.018 \| 1.7126949 \| 1.06E-119 \| \| *TIE1* \| Vasc_Endo2 \| 1.28 \| 0.429 \| 0.019 \| 2.17713341 \| 6.09E-113 \| \| *TIE1* \| Lymphatic_Endo \| 1.30 \| 0.253 \| 0.019 \| 1.67740096 \| 5.37E-85 \| \| *TEK* \| Schlemm_Endo \| 1.92 \| 0.732 \| 0.045 \| 5.36683928 \| 1.20E-287 \| \| *TEK* \| Vasc_Endo1 \| 1.63 \| 0.66 \| 0.044 \| 3.70607258 \| 3.47E-279 \| \| *TEK* \| Vasc_Endo2 \| 1.81 \| 0.72 \| 0.047 \| 4.71956597 \| 3.71E-231 \| \| *TEK* \| Lymphatic_Endo \| 1.54 \| 0.26 \| 0.049 \| 1.79760929 \| 9.01E-54 \| |
| --- | --- | --- | --- | --- | --- | --- | --- | --- | --- | --- | --- | --- | --- | --- | --- | --- | --- | --- | --- | --- | --- | --- | --- | --- | --- | --- | --- | --- | --- | --- | --- | --- | --- | --- | --- | --- | --- | --- | --- | --- | --- | --- | --- | --- | --- | --- | --- | --- | --- | --- | --- | --- | --- | --- | --- | --- | --- | --- | --- | --- | --- | --- | --- |

***Differential gene expression P-value was adjusted with Benjamini-Hochberg method to correct for multiple hypothesis testing.**
