# Supplementary material for: TIE1 and TEK signalling, intraocular pressure, and primary open-angle glaucoma: a Mendelian randomization study
Source: J Transl Med. 2023 Nov 24;21:847. doi: 10.1186/s12967-023-04737-9 (PMC10668387; doi:10.1186/s12967-023-04737-9)
Supplement: Supplementary file 14 — Additional file 14: Table S14. List of cell types identified in six anterior segment tissues from healthy human eyes based on clustering of single-nucleus RNA-sequencing data. [file 12967_2023_4737_MOESM14_ESM.docx]

**Table S14. List of cell types identified in six anterior segment tissues from healthy human eyes based on clustering of single-nucleus RNA-sequencing data**

| **Tissue** | **Cell type abbreviation** | **Cell type description** |
| --- | --- | --- |
| Anterior_Segment | K_Fibro | corneal fibroblasts |
| Anterior_Segment | Ciliary_Fibro | ciliary fibroblasts |
| Anterior_Segment | K_Endo | corneal endothelium |
| Anterior_Segment | Lens_AnteriorEpi | lens anterior epithelium |
| Anterior_Segment | Iris_Fibro | iris fibroblasts |
| Anterior_Segment | FibroX | fibroblast X (derived from corneoscleral wedge) |
| Anterior_Segment | TM_Fibro | trabecular meshwork fibroblasts |
| Anterior_Segment | Mast | mast cells |
| Anterior_Segment | Scleral_Fibro | scleral fibroblasts |
| Anterior_Segment | Lens_EarlyFiber | lens early fiber |
| Anterior_Segment | Lens_EquatorEpi | lens equator epithelium |
| Anterior_Segment | Pericyte1 | pericytes cluster 1 |
| Anterior_Segment | Iris_Sphincter | iris Sphincter muscle cells |
| Anterior_Segment | Ciliary_Muscle | ciliary muscle |
| Anterior_Segment | K_Epi-Wing2 | corneal wing epithelium cluster 2 |
| Anterior_Segment | Schwann | Schwann cells |
| Anterior_Segment | Pericyte2 | pericytes cluster 2 |
| Anterior_Segment | Conj_Epi-Basal | conjunctival basal epithelium |
| Anterior_Segment | K_Epi-Superficial | corneal superficial epithelium |
| Anterior_Segment | Vasc_Endo1 | vascular endothelium cluster 1 |
| Anterior_Segment | Goblet | goblet cells |
| Anterior_Segment | Conj_Epi-Superficial | conjunctival superficial epithelium |
| Anterior_Segment | M√∏ | macrophages |
| Anterior_Segment | Lens_Fiber | lens fiber |
| Anterior_Segment | Lens_GerminativeEpi | lens germinative epithelium |
| Anterior_Segment | Iris_APE | iris anterior pigmented epithelium |
| Anterior_Segment | CB_NPCE | ciliary body non-pigmented ciliary epithelium |
| Anterior_Segment | Iris_PPE | iris posterior pigmented epithelium |
| Anterior_Segment | CB_PCE | ciliary body pigmented ciliary epithelium |
| Anterior_Segment | Uveal_Melanocyte | uveal melanocytes |
| Anterior_Segment | K_Conj_Epi-Superficial | corneal conjunctival superficial epithelium |
| Anterior_Segment | K_Epi-Basal | corneal basal epithelium |
| Anterior_Segment | K_Epi-Wing1 | corneal wing epithelium cluster 1 |
| Anterior_Segment | Conj_Melanocyte | conjunctival melanocytes |
| Anterior_Segment | Vasc_Endo2 | vascular endothelium cluster 2 |
| Anterior_Segment | Lymphatic_Endo | lymphatic endothelium |
| Anterior_Segment | Conj_Epi-Wing | conjunctival wing epithelium |
| Anterior_Segment | Schlemm_Endo | Schlemm's canal endothelium |
| Anterior_Segment | Lymphocyte | lymphocytes |
