# Supplementary material for: TIE1 and TEK signalling, intraocular pressure, and primary open-angle glaucoma: a Mendelian randomization study
Source: J Transl Med. 2023 Nov 24;21:847. doi: 10.1186/s12967-023-04737-9 (PMC10668387; doi:10.1186/s12967-023-04737-9)
Supplement: Supplementary file 15 — Additional file 15: Table S15. STROBE-MR Checklist. [file 12967_2023_4737_MOESM15_ESM.docx]

**Table S15 - STROBE-MR Checklist**

| **Item No.** | **Section** | **Checklist item** | **Relevant text from manuscript** |
| --- | --- | --- | --- |
| 1 | **TITLE and ABSTRACT** | Indicate Mendelian randomization (MR) as the study’s design in the title and/or the abstract if that is a main purpose of the study | *See Title - TIE1/TEK signalling, intraocular pressure, and primary open-angle glaucoma: a Mendelian randomization and colocalization study* |
|  | **INTRODUCTION** |  |  |
| 2 | **Background** | Explain the scientific background and rationale for the reported study. What is the exposure? Is a potential causal relationship between exposure and outcome plausible? Justify why MR is a helpful method to address the study question | *See Introduction – TIE1/TEK signalling in SC has been proposed as a potential therapeutic target in the treatment of elevated IOP. TIE1 and TEK (also known as TIE2) are transmembrane protein receptor tyrosine kinases that are highly expressed in SC endothelial cells. Given the high false discovery rate of animal studies in predicting drug target efficacy in humans, human-centric genetic support for TIE1/TEK signalling should be considered critical in its prioritization as a drug target worth pursuing in large-scale randomized controlled trials (RCTs).* |
| 3 | **Objectives** | State specific objectives clearly, including pre-specified causal hypotheses (if any). State that MR is a method that, under specific assumptions, intends to estimate causal effects | *See Introduction – Drug target Mendelian randomization leverages naturally arising human genetic variation to infer the causal effect of a putative drug target on an outcome. Thus, given the inherent vulnerability of conventional observational studies to unmeasured confounding and reverse causation, MR can strengthen causal inferences made from observational data.*  *See Methods – Drug target MR relies on three core assumptions.* |
|  | **METHODS** |  |  |
| 4 | **Study design and data sources** | Present key elements of the study design early in the article. Consider including a table listing sources of data for all phases of the study. For each data source contributing to the analysis, describe the following: | *See Methods – Figure 1, Data Sources and Table S1* |
|  | a) | Setting: Describe the study design and the underlying population, if possible. Describe the setting, locations, and relevant dates, including periods of recruitment, exposure, follow-up, and data collection, when available. | *See Table S1.*  *Further details about the methodology of the original GWASs can be sought from the original publications, which are referenced accordingly.* |
|  | b) | Participants: Give the eligibility criteria, and the sources and methods of selection of participants. Report the sample size, and whether any power or sample size calculations were carried out prior to the main analysis | *See Methods – Data Sources and Table S1.*  *Given the use of prior GWAS data for exposures and outcomes, MR power calculations are not truly a priori. Furthermore, one requirement of a power calculation is the change in IOP or risk of POAG per SD change in TIE1/ TEK signalling based on previous observational studies, but no such data exist. Thus, power calculations were not considered appropriate.* |
|  | c) | Describe measurement, quality control and selection of genetic variants | *See Methods – Genetic Instruments and Table S1* |
|  | d) | For each exposure, outcome, and other relevant variables, describe methods of assessment and diagnostic criteria for diseases | *See Table S1.* |
|  | e) | Provide details of ethics committee approval and participant informed consent, if relevant | *See Methods – Data Sources - Informed consent for all participants was obtained in the original studies, which were granted relevant ethical approval.* |
| 5 | **Assumptions** | Explicitly state the three core IV assumptions for the main analysis (relevance, independence and exclusion restriction) as well as assumptions for any additional or sensitivity analysis | *See Methods – Drug target MR relies on three core assumptions. First, the genetic instrument associates robustly with the drug target (relevance). Second, the genetic instrument shares no common cause with the outcome (independence). Third, the genetic instrument influences the outcome only via its effect on the drug target (exclusion-restriction). These are illustrated in Figure 2.*  *Assumptions of pleiotropy-robust sensitivity analysis are explained in the Supplementary Methods.* |
| 6 | **Statistical**  **methods: main analysis** | Describe statistical methods and statistics used |  |
|  | a) | Describe how quantitative variables were handled in the analyses (i.e., scale, units, model) | *See Methods – MR estimates represent the change in IOP and odds ratio for POAG, per SD increase in sTIE1 and per SD decrease in sTEK.* |
|  | b) | Describe how genetic variants were handled in the analyses and, if applicable, how their weights were selected | *See Methods – Genetic associations were harmonised by aligning effect alleles in both exposure and outcome datasets. Where multiple variants comprised the instrument, the inverse-variance weighted (IVW) method was used to generate the MR effect estimate. The IVW approach assumes the absence of any horizontal pleiotropy, and so methods robust to the influence of pleiotropy and violation of the third MR assumption were used as sensitivity analyses. The weighted-median, Contamination Mixture, MR-Egger and MR-PRESSO methods were applied.* |
|  | c) | Describe the MR estimator (e.g. two-stage least squares, Wald ratio) and related statistics. Detail the included covariates and, in case of two-sample MR, whether the same covariate set was used for adjustment in the two samples | *See Methods – MR estimates were generated by calculating the Wald-ratio = the variant-outcome regression coefficient divided by the variant-exposure regression coefficient.*  *See Table S1. No co-variates adjusted for beyond principal components in either exposure or outcome GWASs.* |
|  | d) | Explain how missing data were addressed | *Not applicable (only summary data used).* |
|  | e) | If applicable, indicate how multiple testing was addressed | *No correction was made for multiple testing* |
| 7 | **Assessment of**  **assumptions** | Describe any methods or prior knowledge used to assess the assumptions or justify their validity | *IV1 - F-statistics were calculated to quantify the strength of the association between the instrument and the exposure. See Table S2.*  *IV3 - The IVW approach assumes the absence of any horizontal pleiotropy, and so methods robust to the influence of pleiotropy and violation of the third MR assumption were used as sensitivity analyses. The weighted-median, Contamination Mixture, MR-Egger and MR-PRESSO methods were applied. Further details of these methods can be found in the Supplementary Materials.* |
| 8 | **Sensitivity analyses and additional analyses** | Describe any sensitivity analyses or additional analyses performed (e.g. comparison of effect estimates from different approaches, independent replication, bias analytic techniques, validation of instruments, simulations) | *See Methods - Sensitivity analyses for pleiotropy, replication using an independent exposure GWAS for TIE1 and TEK pQTLs, colocalization and single-cell transcriptomic differential gene expression analysis.* |
| 9 | **Software and preregistration** |  |  |
|  | a) | Name statistical software and package(s), including version and settings used | *Analyses were performed using the TwoSampleMR, MendelianRandomization, MR-PRESSO and coloc packages in R (version 4.1.2).* |
|  | b) | State whether the study protocol and details were pre-registered (as well as when and where) | *Study protocol was not pre-registered.* |
|  | **RESULTS** |  |  |
| 10 | **Descriptive data** |  |  |
|  | a) | Report the numbers of individuals at each stage of included studies and reasons for exclusion. Consider use of a flow diagram | *Figure 1 provides flow diagram of study design inc. number of participants. No participants were excluded.* |
|  | b) | Report summary statistics for phenotypic exposure(s), outcome(s), and other relevant variables (e.g., means, SDs, proportions) | *See Supplementary Table S1.* |
|  | c) | If the data sources include meta-analyses of previous studies, provide the assessments of heterogeneity across these studies | *Full details of assessments of heterogeneity can be found in the original publications of the GWASs.*  *.* |
|  | d) | For two-sample MR:   1. Provide justification of the similarity of the genetic variant-exposure associations between the exposure and outcome samples 2. Provide information on the number of individuals who overlap between the exposure and outcome studies | 1. *All data sources comprised exclusively European ancestry individuals.* 2. *There is no overlap between our exposure data taken from the Decode Icelandic cohort and our outcome data. In any case, we calculate F-statistics (see Table S2) showing a low risk of weak instrument bias.* |
| 11 | **Main results** |  |  |
|  | a) | Report the associations between genetic variant and exposure, and between genetic variant and outcome, preferably on an interpretable scale | *See Figures S1 and S2* |
|  | b) | Report MR estimates of the relationship between exposure and outcome, and the measures of uncertainty from the MR analysis, on an interpretable scale, such as odds ratio or relative risk per SD difference | *See Results, Figures 3 and 4* |
|  | c) | If relevant, consider translating estimates of relative risk into absolute risk for a meaningful time period | *Not applicable* |
|  | d) | Consider plots to visualize results (e.g. forest plot, scatterplot of associations between genetic variants and outcome versus between genetic variants and exposure) | *Forest plots are illustrated in Figures 3 and 4 in main manuscript. Leave-one-out plots illustrated in Figures S1 and S2.* |
| 12 | **Assessment of assumptions** |  |  |
|  | a) | Report the assessment of the validity of the assumptions | *Validity of IV assumptions discussed Results and Discussion sections.* |
|  | b) | Report any additional statistics (e.g., assessments of heterogeneity across genetic variants, such as *I^2^*, Q statistic or E-value) | *Calculations of R^2^ and F-statistics discussed in Methods – Genetic Instruments. R^2^ and F-statistics are detailed in Table S2.* |
| 13 | **Sensitivity analyses and additional analyses** |  |  |
|  | a) | Report any sensitivity analyses to assess the robustness of the main results to violations of the assumptions | *See Results discussion of pleiotropy-robust sensitivity analyses, Figures 3 and 4.* |
|  | b) | Report results from other sensitivity analyses or additional analyses | *See Results – Colocalization analysis, Table 1, Figures 5 and 6.*  *See Results - Single cell expression in six tissues in the anterior segment of the eye, Figure 7.* |
|  | c) | Report any assessment of direction of causal relationship (e.g., bidirectional MR) | *Direction of MR estimates stated in Results and Discussion.* |
|  | d) | When relevant, report and compare with estimates from non-MR analyses | *See Discussion for comparison to prior GWAS and pre-clinical evidence.* |
|  | e) | Consider additional plots to visualize results (e.g., leave-one-out analyses) | *Leave-one-out plots illustrated in Figures S1 and S2* |
|  | **DISCUSSION** |  |  |
| 14 | **Key results** | Summarize key results with reference to study objectives | *See Discussion – TIE1 signalling and IOP; TEK signalling and IOP; TIE1, TEK signalling and POAG.* |
| 15 | **Limitations** | Discuss limitations of the study, taking into account the validity of the IV assumptions, other sources of potential bias, and imprecision. Discuss both direction and magnitude of any potential bias and any efforts to address them | *See Discussion - Limitations* |
| 16 | **Interpretation** |  |  |
|  | a) | Meaning: Give a cautious overall interpretation of results in the context of their limitations and in comparison with other studies | *See Discussion - We find novel, human-centric genetic evidence to support increased TIE1/TEK signalling in lowering IOP, with stronger support for TIE1 than TEK.*  *See Conclusion - In this study, we find novel human genetic support for a causal role of both TIE1 and TEK signalling in regulating IOP. Furthermore, Mendelian randomization and colocalization analyses provide stronger support for TIE1 than TEK as a potential IOP-lowering therapeutic target. Further clinical studies investigating this prospect are warranted.* |
|  | b) | Mechanism: Discuss underlying biological mechanisms that could drive a potential causal relationship between the investigated exposure and the outcome, and whether the gene-environment equivalence assumption is reasonable. Use causal language carefully, clarifying that IV estimates may provide causal effects only under certain assumptions | *See Discussion – Molecular interactions between TIE1 and TEK in IOP in SC are discussed, including in relation to ANGPT signalling.* |
|  | c) | Clinical relevance: Discuss whether the results have clinical or public policy relevance, and to what extent they inform effect sizes of possible interventions | *See Discussion – MR estimates represent small lifelong genetic differences in TIE1/TEK signalling perturbation starting and so the magnitude of MR estimates are not interpretable on the same scale as those of a discrete clinical intervention, e.g., estimates derived from an RCT investigating TIE1/TEK signalling perturbation.*  *See Conclusion - Further clinical studies investigating this prospect are warranted.* |
| 17 | **Generalizability** | Discuss the generalizability of the study results (a) to other populations, (b) across other exposure periods/timings, and (c) across other levels of exposure | *See Discussion – Limitations*  *Restricting analysis to European ancestry individuals to avoid confounding by ancestry means these findings may not be generalisable to other ancestries.* |
|  | **OTHER INFORMATION** |  |  |
| 18 | **Funding** | Describe sources of funding and the role of funders in the present study and, if applicable, sources of funding for the databases and original study or studies on which the present study is based | *See Acknowledgements – Funding/ Support.* |
| 19 | **Data and data sharing** | Provide the data used to perform all analyses or report where and how the data can be accessed, and reference these sources in the article. Provide the statistical code needed to reproduce the results in the article, or report whether the code is publicly accessible and if so, where | *TIE1 and TEK exposure GWAS data and POAG outcome data are publicly available. IOP outcome data are not currently publicly available.*  *Code for analyses conducted with TwoSampleMR and MendelianRandomization packages in R (version 4.1.2) are publicly available at* [*https://github.com/MRCIEU/TwoSampleMR*](https://github.com/MRCIEU/TwoSampleMR) *and* [*https://github.com/cran/MendelianRandomization*](https://github.com/cran/MendelianRandomization)*.* |
| 20 | **Conflicts of Interest** | All authors should declare all potential conflicts of interest | *See Acknowledgements - Conflicts of interest disclosures* |
