# Supplementary material for: TIE1 and TEK signalling, intraocular pressure, and primary open-angle glaucoma: a Mendelian randomization study
Source: J Transl Med. 2023 Nov 24;21:847. doi: 10.1186/s12967-023-04737-9 (PMC10668387; doi:10.1186/s12967-023-04737-9)
Supplement: Supplementary file 16 — Additional file 16: Figure S1. Leave-one-out plot for MR estimate of genetically proxied TIE1 and IOP. [file 12967_2023_4737_MOESM16_ESM.pdf]

Figure S1 – Leave-one-out plot for MR estimate of genetically proxied TIE1 and IOP

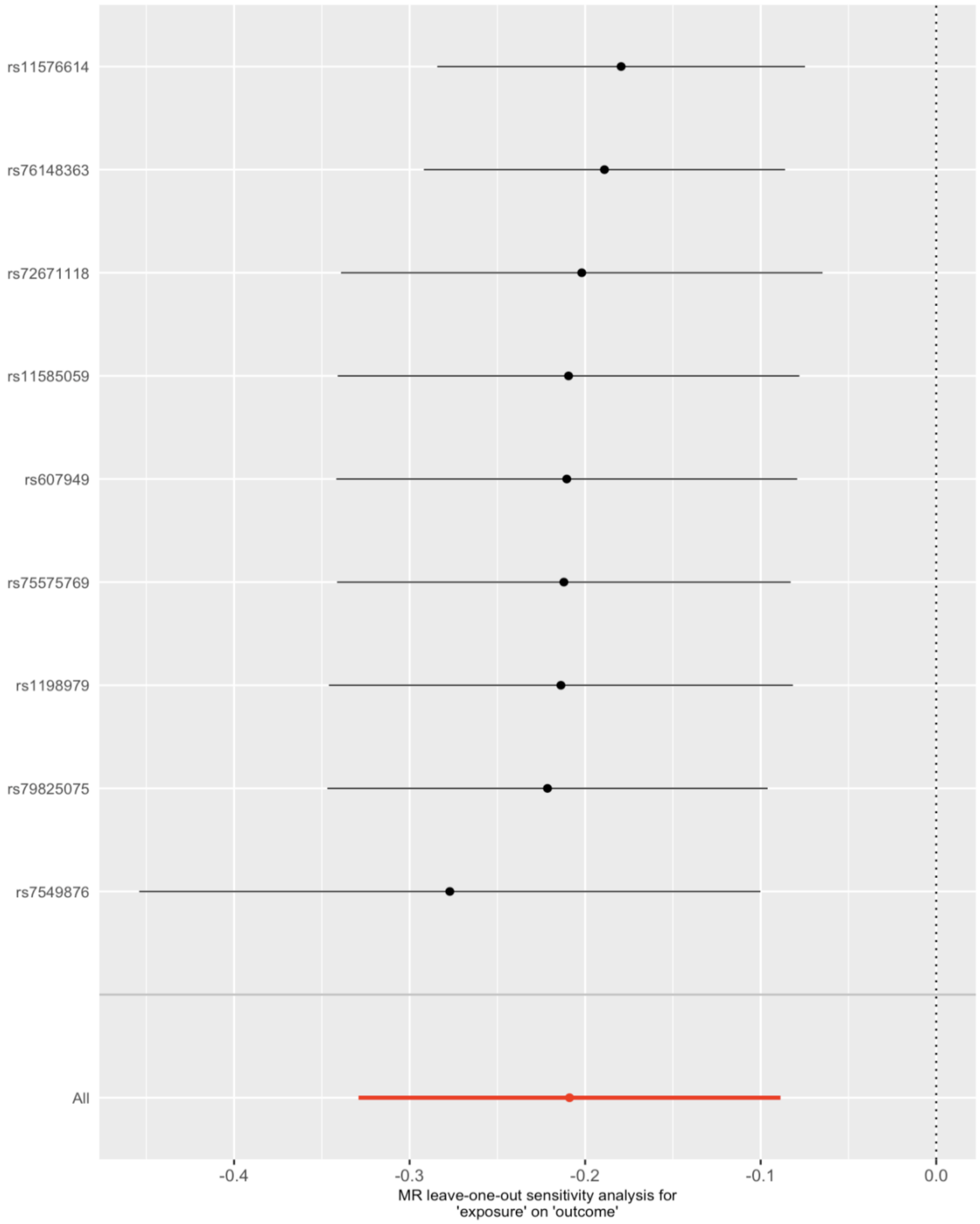

Exposure = increased genetically proxied TIE1 signalling; Outcome = IOP
