# Supplementary material for: TIE1 and TEK signalling, intraocular pressure, and primary open-angle glaucoma: a Mendelian randomization study
Source: J Transl Med. 2023 Nov 24;21:847. doi: 10.1186/s12967-023-04737-9 (PMC10668387; doi:10.1186/s12967-023-04737-9)

Figure S3 – Scatter plot of the genetic associations of instrumental variants with sTIE1 and intraocular pressure.

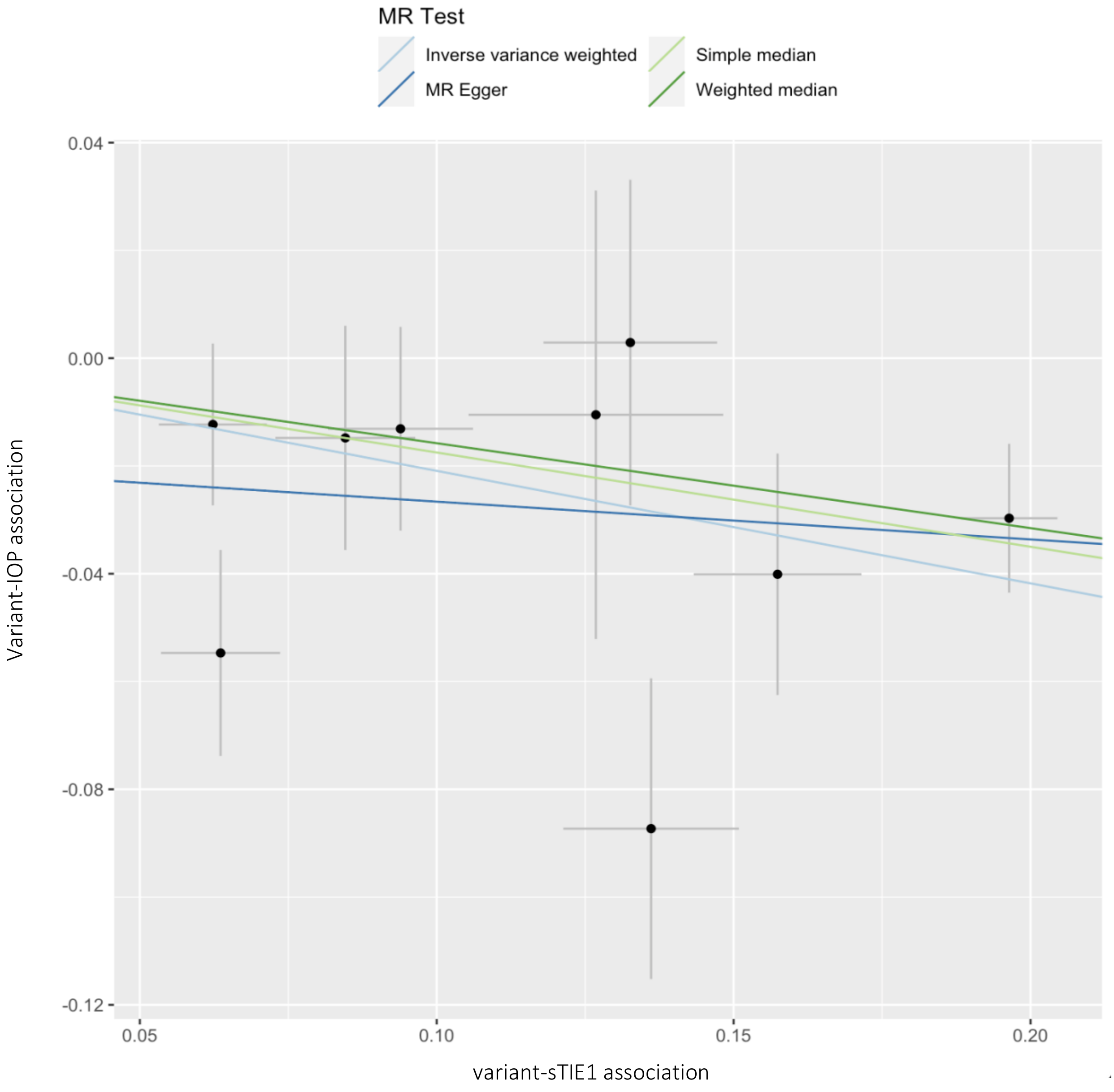

Supplement: Supplementary file 18 — Additional file 18: Figure S3. Scatter plot of the genetic associations of instrumental variants with sTIE1 and intraocular pressure. [file 12967_2023_4737_MOESM18_ESM.pdf]
