# Supplementary material for: TIE1 and TEK signalling, intraocular pressure, and primary open-angle glaucoma: a Mendelian randomization study
Source: J Transl Med. 2023 Nov 24;21:847. doi: 10.1186/s12967-023-04737-9 (PMC10668387; doi:10.1186/s12967-023-04737-9)

Figure S4 – Scatter plot of the genetic associations of instrumental variants with sTEK and intraocular pressure.

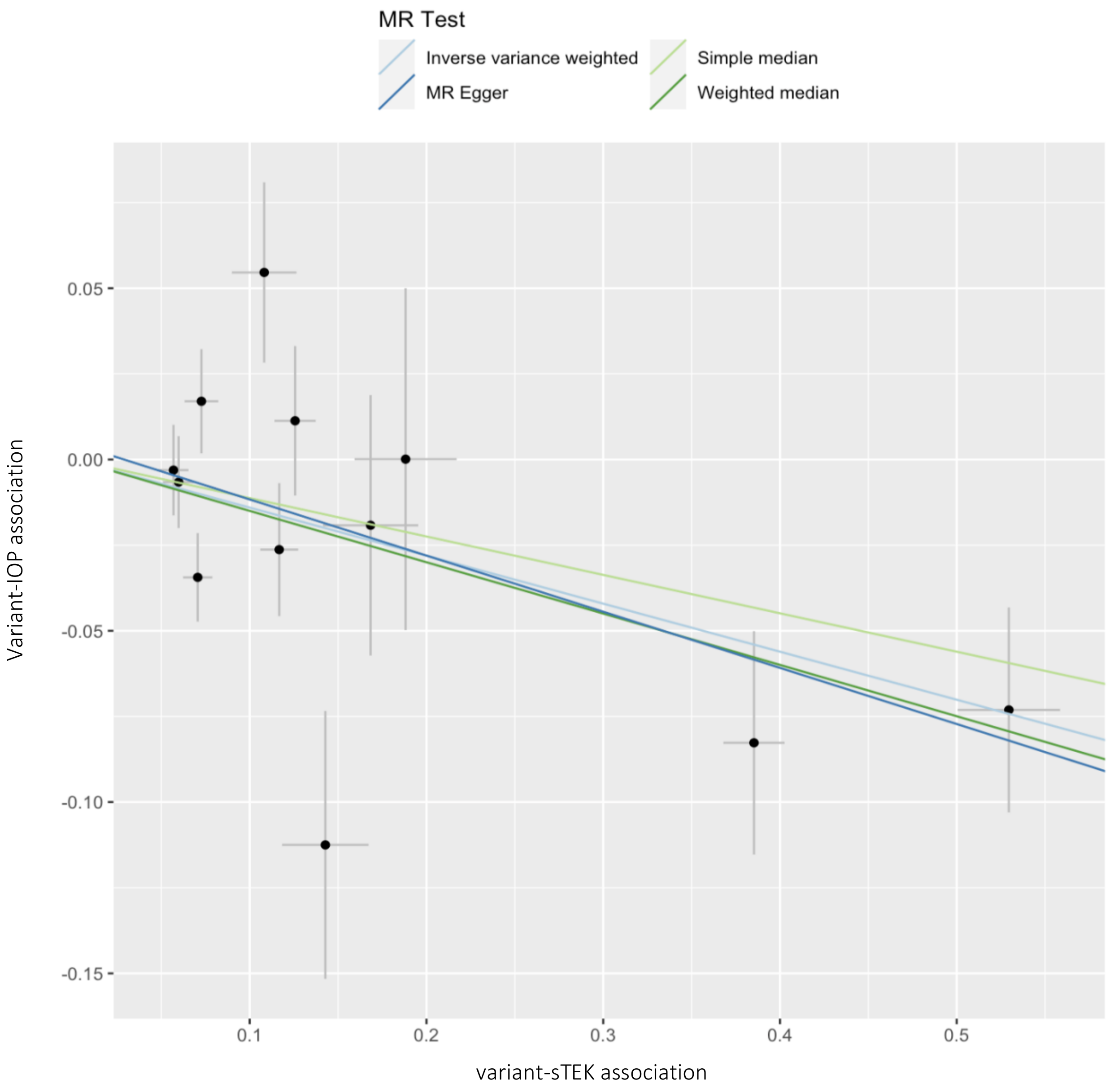

Supplement: Supplementary file 19 — Additional file 19: Figure S4. Scatter plot of the genetic associations of instrumental variants with sTEK and intraocular pressure. [file 12967_2023_4737_MOESM19_ESM.pdf]
