# Supplementary material for: TIE1 and TEK signalling, intraocular pressure, and primary open-angle glaucoma: a Mendelian randomization study
Source: J Transl Med. 2023 Nov 24;21:847. doi: 10.1186/s12967-023-04737-9 (PMC10668387; doi:10.1186/s12967-023-04737-9)
Supplement: Supplementary file 20 — Additional file 20: Figure S5. LocusZoom plot of genetic associations with sTEK protein levels and IOP in TEK gene region. [file 12967_2023_4737_MOESM20_ESM.pdf]

Figure S5 – LocusZoom plot of genetic associations with sTEK protein levels and IOP in *TEK* gene region.

A

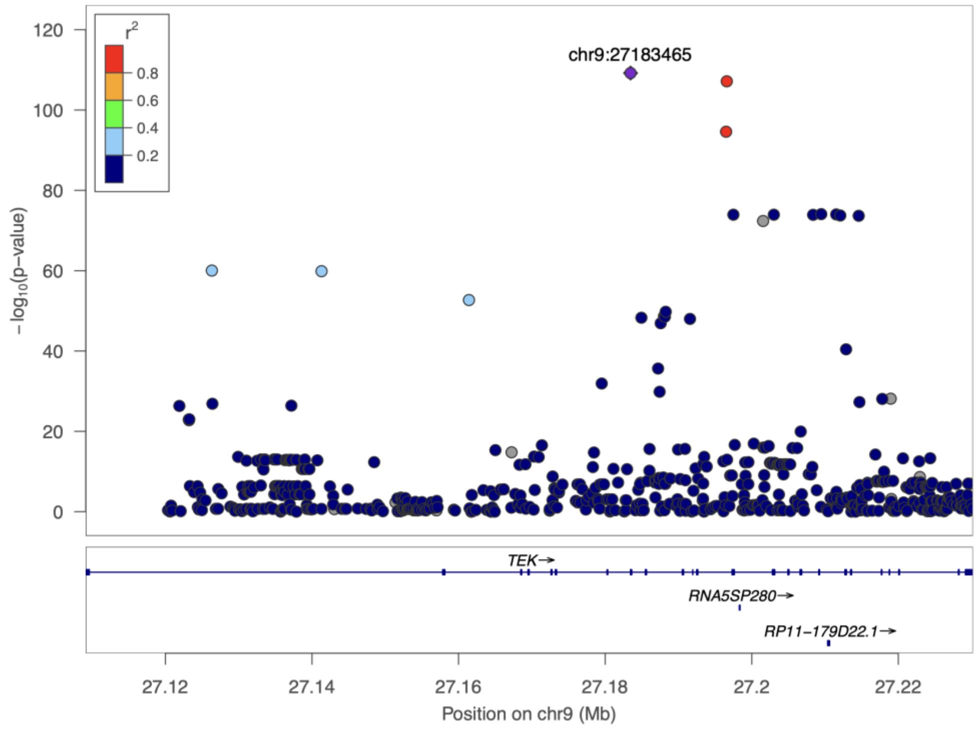

B

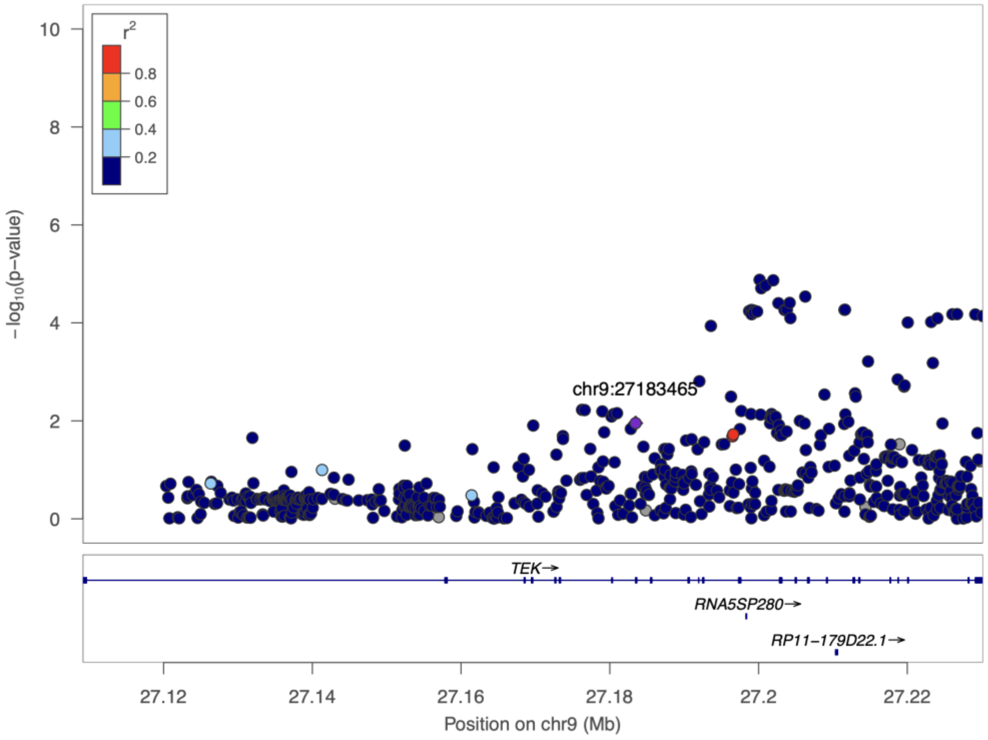

LocusZoom plot of genetic associations in *TEK* gene region with circulating sTEK protein levels (A) and IOP (B). The purple diamond is the genetic variant with the highest posterior probability of being the shared causal variant underlying the two traits, as determined by colocalization.
