# Supplementary material for: TIE1 and TEK signalling, intraocular pressure, and primary open-angle glaucoma: a Mendelian randomization study
Source: J Transl Med. 2023 Nov 24;21:847. doi: 10.1186/s12967-023-04737-9 (PMC10668387; doi:10.1186/s12967-023-04737-9)
Supplement: Supplementary file 21 — Additional file 21: Figure S6. LocusCompare plot of genetic associations with sTEK protein levels and IOP in the TEK gene region. [file 12967_2023_4737_MOESM21_ESM.pdf]

**Figure S6 – LocusCompare plot of genetic associations with sTEK protein levels and IOP in the *TEK* gene region.**

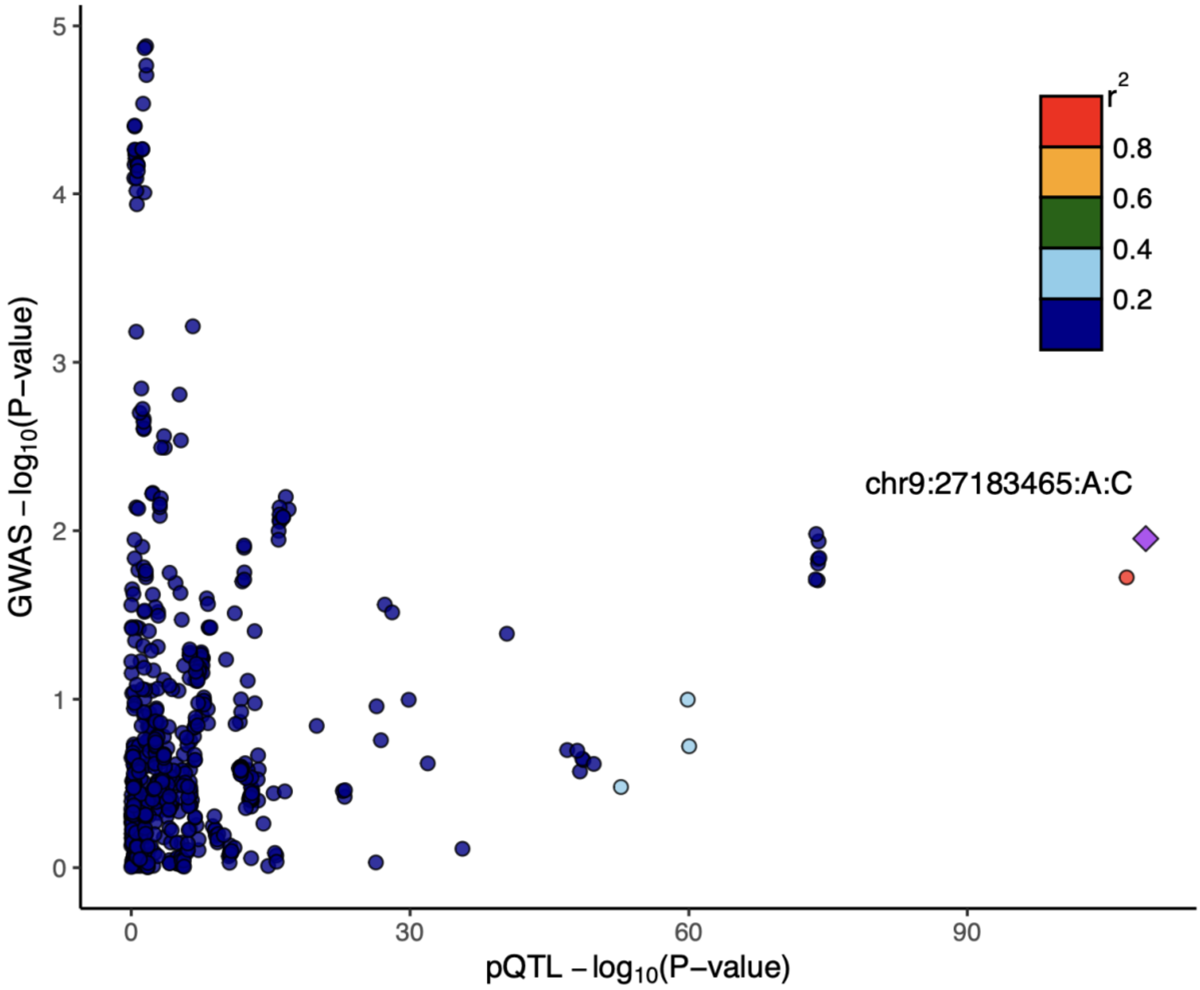


LocusCompare plot illustrating genetic associations within *TEK* gene region with circulating sTEK protein levels (x-axis) and IOP (y-axis). The purple diamond is the genetic variant with the strongest association with siEK2 protein levels. Points are color-coded based on each variant's LD ($r^2$) relative to the variant with the highest colocalization posterior probability in the gene region. Based on the observed correlation pattern, there seem to be two independent haplotypes in the IOP GWAS locus, with the sTEK2 pQTL correlating with the haplotype with more modest IOP association p-values.
