# Supplementary material for: TIE1 and TEK signalling, intraocular pressure, and primary open-angle glaucoma: a Mendelian randomization study
Source: J Transl Med. 2023 Nov 24;21:847. doi: 10.1186/s12967-023-04737-9 (PMC10668387; doi:10.1186/s12967-023-04737-9)
Supplement: Supplementary file 22 — Additional file 22: Figure S7. LocusZoom plot of genetic associations with sTIE1 protein levels in the TIE1 gene region with 100kB window. [file 12967_2023_4737_MOESM22_ESM.pdf]

Figure S7 – LocusZoom plot of genetic associations with sTIE1 protein levels in the *TIE1* gene region with 100kB window

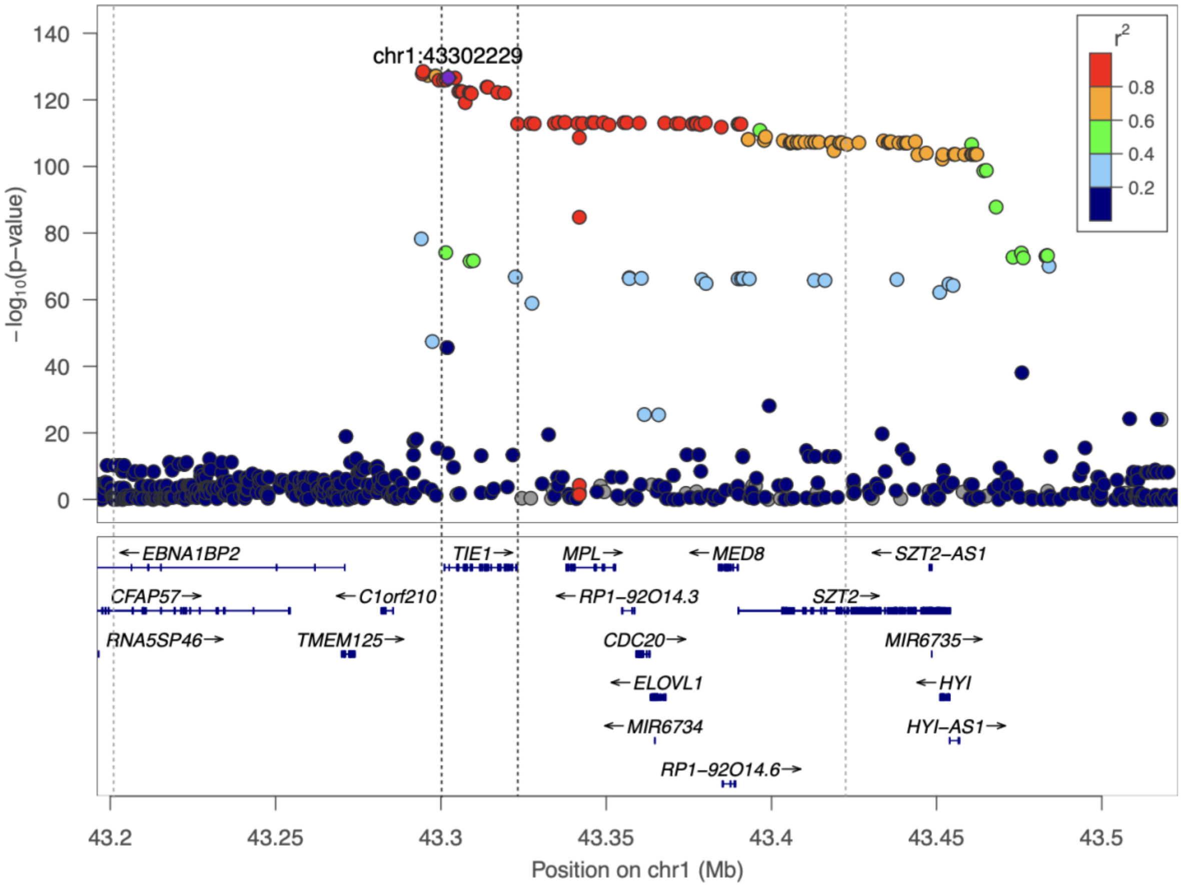

LocusZoom plot of genetic associations in *TIE1* gene region with circulating sTIE1 protein levels. The purple diamond is the genetic variant with the highest posterior probability of being the shared causal variant underlying the two traits, as determined by colocalization. Dotted grey lines indicate boundaries of *TIE1* gene body and 100kB window either side.
