# Supplementary material for: TIE1 and TEK signalling, intraocular pressure, and primary open-angle glaucoma: a Mendelian randomization study
Source: J Transl Med. 2023 Nov 24;21:847. doi: 10.1186/s12967-023-04737-9 (PMC10668387; doi:10.1186/s12967-023-04737-9)
Supplement: Supplementary file 23 — Additional file 23: Methods S1. Supplementary Methods. [file 12967_2023_4737_MOESM23_ESM.docx]

**Additional Methods**

**Pleiotropy-robust Sensitivity Analyses**

Horizontal pleiotropy is where the genetic instrument influences the outcome not exclusively via the exposure and its presence violates the third MR assumption (exclusion-restriction). The IVW approach provides a valid causal estimate under the assumption that all variants are valid instruments [1, 2], i.e., that no variants are pleiotropic or that pleiotropy is balanced.

In median-based methods, a median rather than mean of the Wald estimates is calculated. Provided that over 50% of variants are valid instruments, median-based estimates will produce valid causal estimates. In the simple-median approach [3], all variant-specific estimates are weighted equally, whereas in the weighted-median approach [3], variants are weighted inversely to their standard errors.

The contamination mixture method provides consistent causal estimates where a plurality of the genetic variants are valid instruments [4]. A likelihood function is constructed from the variant-specific ratio estimates. If a variant is a valid instrument, then its ratio estimate is assumed to be normally distributed about the true causal effect. If a variant is an invalid instrument, then its ratio estimate is assumed to be distributed about 0 with a large standard deviation. The likelihood is then maximised over different values of the causal effect and different configurations of valid and invalid instruments. Confidence intervals are usually not symmetric. Default parameters were used.

Rather than fixing the intercept at the origin, as done in the IVW approach, MR-Egger [5] allows the intercept to float and provides a valid causal estimate in the presence of directional pleiotropy. MR-Egger requires the instrument strength independent of the direct effects (INSIDE) assumption, i.e., it requires that pleiotropic effects of the instrumental variants are independent of the association between the variants and the exposure. The precision of MR-Egger depends on the variance between genetic associations with the exposure and so if the different instrumental variants are similarly associated with the exposure, MR-Egger will be imprecise and have wide confidence intervals. Accordingly, of the different sensitivity analyses used, MR-Egger can be expected to have the widest confidence intervals. Via the Egger intercept significance test, the deviation of the intercept term from the origin indicates whether significant directional pleiotropy is present.

The MR-Pleiotropy Residual Sum and Outlier (MR-PRESSO) method [6] takes the residual sum of squares from the IVW linear regression as a measure of heterogeneity. The IVW method is performed iteratively, removing each instrumental variant in turn and the RSS calculated for each omission. Significantly heterogenous variants are removed and the IVW method used to calculate the MR estimate using the remaining variants.

**Colocalization**

Genetic colocalization assesses the likelihood that two (or more) phenotypic traits, such as an exposure and outcome, share the same causal variant as opposed to distinct causal variants [7] [8] [9].

In recent years, colocalization has increasingly been employed as a supplementary analysis to MR, especially in drug-target MR studies [10] [11]. Performed where MR yields an apparently significant effect of a putative drug target and outcome of interest, colocalization can interrogate the risk that this MR result is confounded by linkage disequilibrium (LD). Here two SNPs in the drug target gene region of interest are correlated with one another, with one SNP associating with the exposure and the other SNP associating with outcome. On performing MR in this situation, the genetic instrument may appear to associate with both exposure and outcome and produce a significant MR result when in fact two distinct causal variants in LD with one another independently explain the two associations with exposure and outcome. By formally assessing the likelihood that a single variant or distinct variants explain the association with exposure and outcome, colocalization can identify confounding by LD and thus identify spurious MR results.

Two main theoretical approaches to colocalization have been developed: a frequentist, proportional approach and a Bayesian, enumeration approach [7] [8] [9]. A limitation of proportional, frequentist approach is that colocalization is concluded to be present when the null holds. However, failure to reject the null may be due to true colocalization or a lack of statistical power and proportional approaches are incapable of distinguishing between the two [9]. A key advantage of enumeration methods is that colocalization is only concluded in the presence of positive evidence supporting colocalization. In the absence of evidence of colocalization, the posterior probabilities will approximate the prior probabilities, which are intentionally set at sceptical values to avoid spurious false positives. Coloc, the most widely used enumeration method, was used in this study [7].

Using the prior probabilities and the genetic association data for the two traits within the drug target gene body, coloc was used to compute posterior probabilities for five hypotheses: H0: no association with either trait; H1: association with trait 1, but not trait 2; H2: association with trait 2, but not trait 1; H3: association with both traits, but at separate causal variants; H4: association with both traits at a shared causal variant.

**Replication**

For replication, the parameters for *cis-*MR and coloc analyses were unchanged, i.e., the same *P*-value, LD clumping threshold and 100kB window were used in the *cis-*MR analysis, and the same priors (*p*1, *p*2 and *p*12) in the coloc analysis.

# References

| [1] | Burgess S, Small DS, Thompson SG. A review of instrumental variable estimators for Mendelian randomization. Stat Methods Med Res. 2017;26(5):2333-2355. |
| --- | --- |
| [2] | Burgess S, Butterworth A, Thompson SG. Mendelian randomization analysis with multiple genetic variants using summarized data. Genet Epidemiol. 2013;37(7):658-665. |
| [3] | Bowden J, Davey Smith G, Haycock PC, Burgess S. Consistent Estimation in Mendelian Randomization with Some Invalid Instruments Using a Weighted Median Estimator. Genet Epidemiol. 2016;40(4):304-314. |
| [4] | Burgess S, Foley CN, Allara E, Staley JR, Howson JMM. A robust and efficient method for Mendelian randomization with hundreds of genetic variants. Nat Commun. 2020;11(1):376. |
| [5] | Bowden J, Davey Smith G, Burgess S. Mendelian randomization with invalid instruments: effect estimation and bias detection through Egger regression. Int J Epidemiol. 2015;44(2):512-525. |
| [6] | Verbanck M, Chen CY, Neale B, Do R. Detection of widespread horizontal pleiotropy in causal relationships inferred from Mendelian randomization between complex traits and diseases. Nat Genet. 2018;50(5):693-698. |
| [7] | Giambartolomei C, Vukcevic D, Schadt EE, et al. Bayesian test for colocalisation between pairs of genetic association studies using summary statistics. PLoS Genet. 2014;10(5):e1004383. |
| [8] | Wallace C. A more accurate method for colocalisation analysis allowing for multiple causal variants. PLoS Genet. 2021;17(9):e1009440. |
| [9] | Zuber V, Grinberg NF, Gill D, et al. Combining evidence from Mendelian randomization and colocalization: Review and comparison of approaches. Am J Hum Genet. 2022;109(5):767-782. |
| [10] | Gill D, Georgakis MK, Walker VM, et al. Mendelian randomization for studying the effects of perturbing drug targets. Wellcome Open Res. 2021;6:16. |
| [11] | Wallace C. Eliciting priors and relaxing the single causal variant assumption in colocalisation analyses. PLoS Genet. 2020;16(4):e1008720. |
